# Annexin A2 binds to endosomes and negatively regulates TLR4-triggered inflammatory responses via the TRAM-TRIF pathway

**DOI:** 10.1038/srep15859

**Published:** 2015-11-03

**Authors:** Shuang Zhang, Min Yu, Qiang Guo, Rongpeng Li, Guobo Li, Shirui Tan, Xuefeng Li, Yuquan Wei, Min Wu

**Affiliations:** 1 Department of Basic Sciences, University of North Dakota, Grand Forks, North Dakota 58203-9037, USA; 2 State Key Laboratory of Biotherapy and Cancer Center/Collaborative Innovation Center of Biotherapy, West China Hospital, Sichuan University, Chengdu 610041, China; 3 Department of thoracic oncology, West China Hospital, Sichuan University, Chengdu 610041, China; 4 Department of Rheumatology, Renji Hospital, Shanghai Jiaotong University, Shanghai 200127, China; 5 College of Biotechnology and Pharmaceutical Engineering, Nanjing University of Technology, Nanjing 211800, China; 6 Laboratory of Biochemistry and Molecular Biology, School of Life Sciences, Yunnan University, Kunming 650091, China

## Abstract

Lipopolysaccharide (LPS) derived from Gram-negative bacteria activates plasma membrane signaling via Toll-like receptor 4 (TLR4) on host cells and triggers innate inflammatory responses, but the underlying mechanisms remain to be fully elucidated. Here we reveal a role for annexin A2 (AnxA2) in host defense against infection as *anxa2*^*−/−*^ mice were highly susceptible to Gram-negative bacteria-induced sepsis with enhanced inflammatory responses. Computing analysis and biochemical experiments identified that constitutive AnxA2 expression facilitated TLR4 internalization and its subsequent translocation into early endosomal membranes. It activated the TRAM-dependent endosomal signaling, leading to the release of anti-inflammatory cytokines. Importantly, AnxA2 deficiency prolonged TLR4-mediated signaling from the plasma membrane, which was attributable to pro-inflammatory cytokine production (IL-6, TNFα and IL-1β). Thus, AnxA2 directly exerted negative regulation of inflammatory responses through TLR4-initiated TRAM-TRIF pathway occurring on endosomes. This study reveals AnxA2 as a critical regulator in infection-initiated inflammation, which protects the host from excessive inflammatory damage.

Toll-like receptor 4 (TLR4) is an evolutionarily conserved molecule expressed by a variety of immune cells including professional antigen-presenting cells, and plays a fundamental role in pathogen recognition and activation of innate immunity. TLR4 can recruit four cytosolic adaptors including TIR domain-containing adaptor protein (TIRAP), myeloid differentiation primary response 88 (MyD88), TIR domain-containing adaptor-inducing IFN-β (TRIF), and TRIF-related adaptor molecule (TRAM). It has become clear that TLR4 can induce signal transduction at diverse locations in the cell, with the cell surface and endosomal membranes being the best-defined sites. TLR4 initiates innate immune responses by activating signaling pathways that depend on the plasmalemmal TIRAP-MyD88 or endosomal TRAM-TRIF adaptor complexes, which consequently induce the production of pro-inflammatory cytokines or IFN-β, respectively^1^,^2^,^3^. Agonist-mediated activation of TLR4 response has been extensively studied; however, antagonist-mediated negative regulatory mechanisms remain to be incompletely elucidated. If negative regulators of anti-inflammatory response are weakened, exceeding activation of pro-inflammatory response by TLR4 signaling may result in inflammatory disorders, such as autoimmune diseases and septic shock^4^,^5^,^6^.

Annexins are calcium-dependent, anionic phospholipid-binding proteins. Through molecular interaction on the plasma membrane surface, annexins help provide cell membrane platforms for recruitment and activation of a number of critical signaling proteins. Like other family members, annexin A2 (AnxA2) is pleiotropic protein and is involved in diverse cellular processes, such as cell motility, endocytosis, fibrinolysis, ion channel formation, and cell matrix interactions^7^,^8^. As AnxA2 is an intracellular protein with demonstrated roles in cytoplasmic membrane-associated processes, it has been implicated in the process of inflammatory events^9^. A previous study demonstrates that AnxA2-S100A10 heterotetrameric complex directly activates human macrophages through TLR4-mediated signaling^10^. It has also been demonstrated that the apoptotic process leads to TLR4 activation as well as up-regulation of AnxA2^11^. However, the underlying mechanism how AnxA2 regulates TLR4-triggered inflammatory response to bacterial infection remains unknown^12^.

Endosomes are sub-cellular organelles, and are associated with catabolism of exogenous and endogenous proteins, down-regulation of surface receptors, and elimination of pathogenic organisms. Endosomes comprise three serial compartments: early endosomes, late endosomes, and recycling endosomes. Molecules internalized from the plasma membrane either enter into lysosomes for degradation following an endosome pathway, or are recycled back to the plasma membrane^13^. Furthermore, some molecules contain the polybasic region, which can bind any phosphatidylinositol molecule and facilitate the trafficking from the plasma membrane into endosomes. For instance, TRAM-TRIF adaptor in TLR4 signaling comprises such motifs and binds to the endosomes to generate anti-inflammatory cytokines^14^.

*Klebsiella pneumoniae* (Kp) is the second most common pathogen of Gram-negative bloodstream infection, which usually arises as a complication of respiratory and gastrointestinal infections^15^. In this study, we set out to investigate the role of AnxA2 in inflammatory response to Kp infection using alveolar and peritoneal macrophages in which AnxA2 was pharmacologically or genetically inactivated. We found that AnxA2 facilitated TLR4 internalization and activated the TRAM-dependent signaling in early endosomal membranes, leading to the release of anti-inflammatory cytokines. Conversely, impaired function of AnxA2 prolonged the residence of TLR4 at the plasma membrane and led to amplified production of pro-inflammatory cytokines via mitogen-activated protein kinase (MAPK) and NF-κB pathways. Our results document a non-canonical function for AnxA2 as a critical regulator of TLR4 pathway in the fine tuning of inflammatory responses.

## Results

### AnxA2 attenuates bacteria-induced pulmonary inflammation

**Figure 1:**
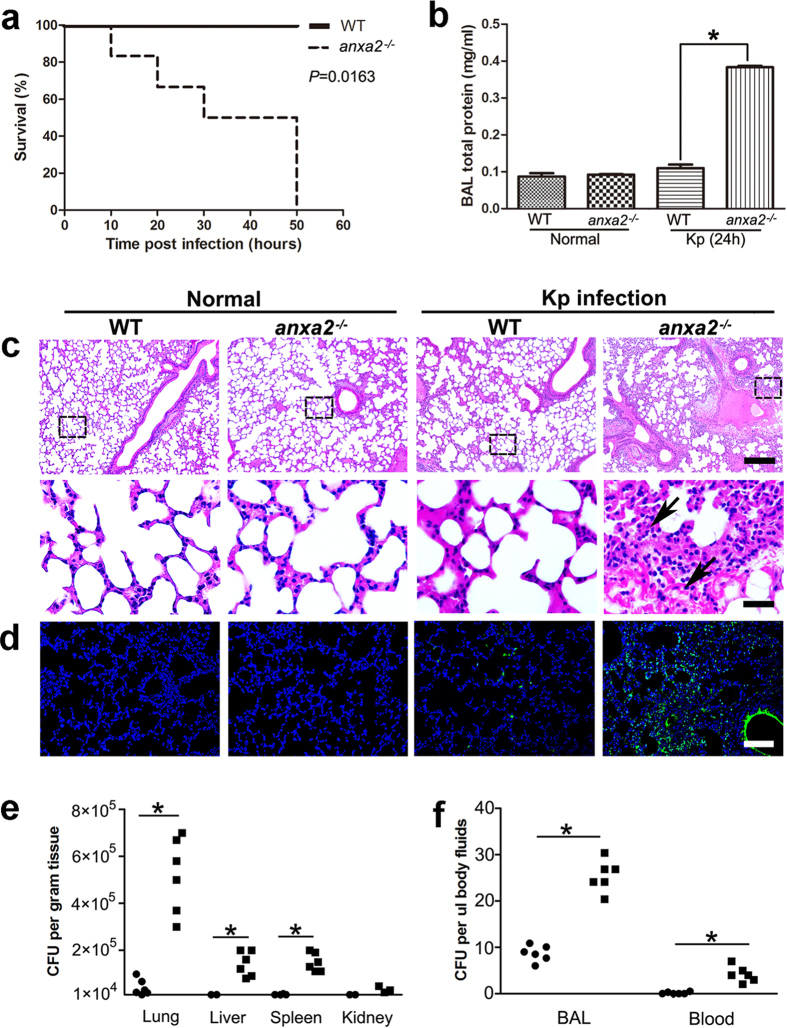
AnxA2 attenuates Kp-induced pulmonary inflammation. *Anxa2*^*−/−*^ and WT mice were intranasally challenged with 1 × 10^5^ CFU of Kp, respectively. (**a**) The mice were maintained up to 60 h. The survival test is represented by Kaplan-Meier survival curves (n = 5; log rank test; *P* = 0.0163). (**b**) 24 h after Kp challenge, total protein concentrations of BAL fluid from *anxa2*^*−/−*^ or WT mice were measured by Bradford assay. Columns, mean; bars, SD (n = 3; ANOVA; *P < 0.05). (**c**) H&E staining of lung tissue sections from WT and *anxa2*^*−/−*^ mice, treated with or without Kp bacteria. Scale bars in upper panels, 200 μm; in lower panels, 20 μm. The lower panels showed relatively clear alveolar structures. Black arrows indicated thickened alveolar interstitium and necrosis in lung tissue of *anxa2*^*−/−*^ mice. (**d**) Lung sections were stained by the TUNEL technique and counterstained with DAPI. TUNEL-positive (green) cells showed *in situ* cell apoptosis in the bronchial and alveolar epithelium. Scale bars, 100 μm. (**e**, **f**) 24 h after infection, bacterial burdens in WT (round dots) and anxa2^*−/−*^ (square dots) mice were quantified. Date were representative of six independent experiments (n = 6; ANOVA; *P < 0.05).

To determine the potential role of AnxA2 in acute pneumonia models, we intranasally instilled Kp (1 × 10^5^ colony-forming units [CFU]/mouse) to *anxa2* knock-out (KO, *anxa2*^*−/−*^) or wild-type (WT) mice. As shown in Fig. 1a, *anxa2*^*−/−*^ mice exhibited increased mortality. At 50 h after infection, all *anxa2*^*−/−*^ mice died, whereas all WT mice remained alive (*P* = 0.0163, log rank test). Consistent with the survival data, *anxa2*^*−/−*^ mice showed evidence of enhanced lung injury with increased protein accumulation (airway leakage) in the bronchoalveolar lavage (BAL) fluid (Fig. 1b), thickened alveolar interstitium (Fig. 1c and Supplementary Fig. 1a), heightened CXC (containing cysteine-X-cysteine motif, X=any amino acid) chemokines and macrophage infiltration (Supplementary Fig. 3a, b), and increased cell apoptosis (Fig. 1d and Supplementary Fig. 1b).

To gauge the underlying molecular events that contributed to these histological alterations, we quantified reactive oxygen species (ROS) in primary macrophages from BAL fluid. After 24 h infection, macrophages of *anxa2*^*−/−*^ mice showed increased oxidative stress as determined by NBT assays (Supplementary Fig. 1c). The intensified oxidation response to Kp infection was further confirmed by dihydro-dichlorofluorescein (H_2_DCF) assay, a sensitive fluorescence method for quantifying superoxide (Supplementary Fig. 1d). Moreover, JC-1 fluorescence assay and MTT assay indicated that increased oxidation may contribute to apoptotic cell death and decreased cell proliferation (Supplementary Fig. 1e, f). In line with the increased oxidative stress, levels of pro-inflammatory cytokines such as IL-1β, IL-6 and TNFα, were higher in local and systemic fluid circulation in mice lacking AnxA2 (Supplementary Fig. 2).

We also quantified bacterial burdens in the lung and several other organs 24 h after Kp infection. Compared to WT mice, *anxa2*^*−/−*^ mice showed significantly increased Kp CFU in the lung, liver, and spleen, as well as BAL fluid and blood, indicating aggressive bacterial expansion, severe lung injury, and systemic spread of infection (Fig. 1e, f). Furthermore, the systemic spread of bacteria may be due to increased vascular permeability in mouse lung tissue (Supplementary Fig. 3c).

### AnxA2 promotes host-mediated intro-abdominal pathogen clearance

**Figure 2:**
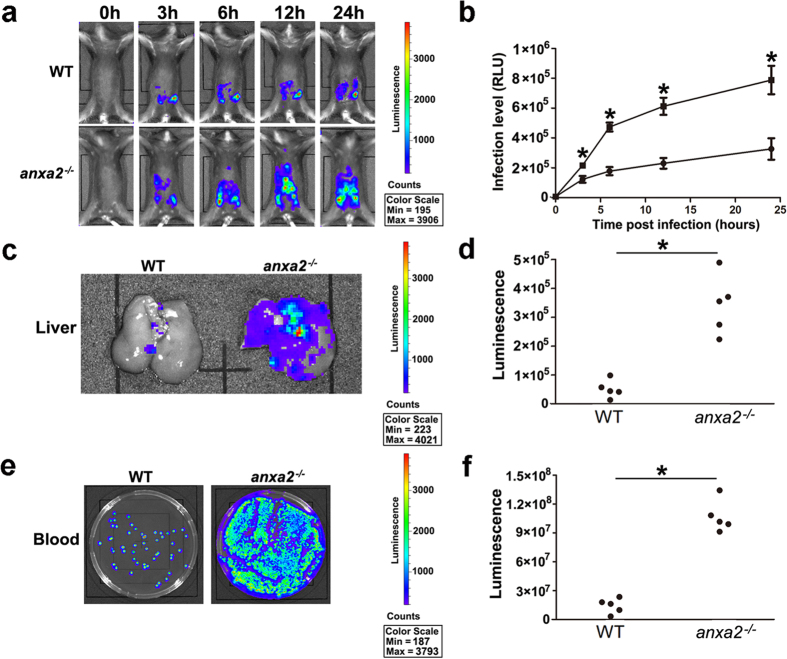
AnxA2 limits Kp spread in peritoneal cavity and circulating blood. Groups of mice (n = 5/group) were anesthetized with ketamine (40 mg/Kg) and imaged at 0, 3, 6, 12 and 24 h after being dosed with 2 × 10^5^ CFU bioluminescent Kp intraperitoneally. (**a**,**b**) Whole animal imaging of bioluminescence was detected by IVIS XRII system at different time points. It illustrated intense luminescence in red, moderate luminescence in green, low-level luminescence in blue, which indicated the severity of infection from strongest to weakest (n = 5; ANOVA; *P < 0.05). (**c**,**d**) 24 h after infection, imaging of extensively washed livers showed enhanced bacterial spread into livers of *anxa2*^*−/−*^ mice (n = 5; ANOVA; *P < 0.05). (**e**,**f**) 24 h after infection, blood plating was cultured overnight and demonstrated a higher bacterial dissemination in the blood of *anxa2*^*−/−*^ mice (n = 5; ANOVA; *P < 0.05).

To discern the pathogenesis in animals, we used a bioluminescent variant of Kp that allowed the *in vivo* monitoring of bacterial growth over time following infection, and heat-killed bioluminescent Kp was served as the control (Supplementary Fig. 3e, f and Supplementary Fig. 4e, f). Each group of mice was inoculated intraperitoneally (i.p.) with a lethal dose of Kp (2 × 10^5^ CFU/mouse). Remarkably, at 24 h post infection, Kp extensively spread in peritoneal cavity of *anxa2*^*−/−*^ mice when compared to WT mice (Fig. 2a, b). The total bioluminescence burdens of the liver and blood were also significantly higher in *anxa2*^*−/−*^ mice at 24 h post infection than those of WT mice (Fig. 2c–f). To confirm this result, we used AnxA2 shRNA lentiviral particles to down-regulate AnxA2 in WT mice by i.p. injection 1 day before Kp infection. While control shRNA lentiviral particles containing a scrambled sequence did not cause specific degradation of mRNA, the AnxA2 shRNA lentiviral particles temporarily decreased AnxA2 in peritoneal macrophages (Supplementary Fig. 4a, b). Importantly, AnxA2 knock down by lentiviruses resulted in wider bacterial dissemination in the peritoneal cavity, liver, and peripheral circulating blood in line with the data from AnxA2 KO mice (Supplementary Fig. 4c,d,g–j). Moreover, the systemic spread of bacteria may be due to increased vascular permeability in peritoneum (Supplementary Fig. 3d). These data collectively indicated that AnxA2 may play a protective role against Kp infection.

### Depleting AnxA2 exacerbates TLR4-triggered inflammatory responses in macrophages

**Figure 3:**
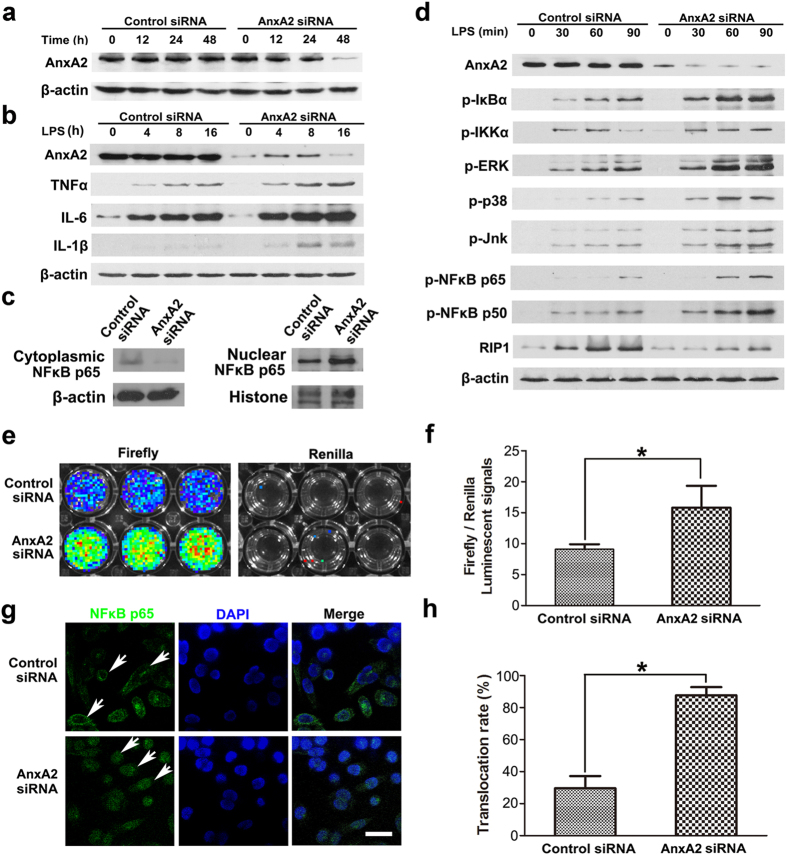
Decreased AnxA2 exacerbates the activation of TLR4 signaling. MH-S cells were transfected with AnxA2 siRNA or control siRNA for 48 h, then stimulated with 100 ng/ml LPS at indicated time points. (**a**) Western blot analysis of AnxA2 in MH-S cells after transfection with control siRNA or siRNA specific for AnxA2. β-actin served as a loading control throughout. (**b**) Western blot analysis of inflammatory cytokines in lysates of macrophages stimulated for 0–16 h with LPS. (**c**) AnxA2 knock-down or normal MH-S cells were stimulated with LPS for 2 h, and NFκB p65 was detected by immunoblotting analysis in cytoplasmic and nuclear fractions from MH-S cells in each group. (**d**) Immunoblotting analysis of phosphorylated signaling molecules in lysates of macrophages stimulated for 0–90 min with LPS. Cropped blots are from gels run under the same experimental condition, and the full-length blots are presented in Supplementary Fig. 9a, b, c, d. (**e**,**f**) AnxA2 knock-down or normal MH-S cells were transiently transfected with pNF-κB-luc plasmid for 24 h, then stimulated with LPS for another 12 h. Cell lysates were subjected to luciferase activity analysis using the Dual-Luciferase Reporter Assay System. The left panel indicated firefly luciferase activity, while the right panel revealed renilla luciferase activity as the control. It was performed in 3 replicates. Columns, mean; bars, SD (n = 3; ANOVA; *P < 0.05). (**g**,**h**) AnxA2 knock-down or normal MH-S cells were stimulated with LPS for 2 h. NFκB translocation was visualized by indirect immunofluorescence staining, and DAPI was used to stain the nucleus. White arrows indicated NFκB p65 cytoplasmic location in normal MH-S cells, and NFκB p65 nuclear location in AnxA2 knock-down MH-S cells. Scale bars, 20 μm. These data were representative of three experiments. Columns, mean; bars, SD (n = 3; ANOVA; *P < 0.05).

To detect whether AnxA2 interacts with inflammatory signaling regulators in macrophages, MH-S alveolar macrophages were used in cell culture assays. MH-S cells were transfected with AnxA2 small interfering RNA (siRNA) and protein expression of AnxA2 was significantly down-regulated at 48 h post-transfection (Fig. 3a). Total RNA was isolated from MH-S cells and reverse transcribed for cDNA microarray analysis, which revealed a number of interesting outcomes. First, it was evident that the decreased AnxA2 levels induced increase in MyD88, which was a critical component of the TLR4-stimulated canonical signaling pathway. Secondly, the co-challenge with AnxA2 siRNA plus LPS led to up-regulated pro-inflammatory gene (IL-6) and down-regulated anti-inflammatory gene (IL-10) (Supplementary Fig. 5). To determine the underlying mechanisms of AnxA2 in inflammatory responses, MH-S cells were transfected with AnxA2 siRNA for 48 h, followed by stimulation with 100 ng/ml LPS at indicated time points. The cell lysates were applied for immunoblotting to detect proteins in MAPK and NF-κB pathways, which are considered downstream signaling proteins of the TLR4 axis. Immunoblotting results showed that TLR4 signaling was significantly activated in LPS-stimulated AnxA2-silenced macrophages. We also observed increased inflammatory cytokine production in LPS-stimulated AnxA2-silenced macrophages in a time dependent manner (Fig. 3b,d). In addition, AnxA2 knockdown dramatically promoted the transcription of NF-κB (Fig. 3e, f). As the translocation of NF-κB was a key step for the initiation of inflammatory responses, we next evaluated the effect of AnxA2 on NF-κB nuclear translocation using immunoblotting and immunofluorescence assay. The results showed that NF-κB was activated and translocated into the nucleus in AnxA2-silenced MH-S cells after LPS stimulation but not seen in control cells (Fig. 3c, g, h). These data suggested that knockdown of AnxA2 enhanced and prolonged TLR4 signaling in macrophages.

### AnxA2 is recruited to a LPS-activated TLR4 complex and controls TLR4 internalization in primary peritoneal macrophages

**Figure 4:**
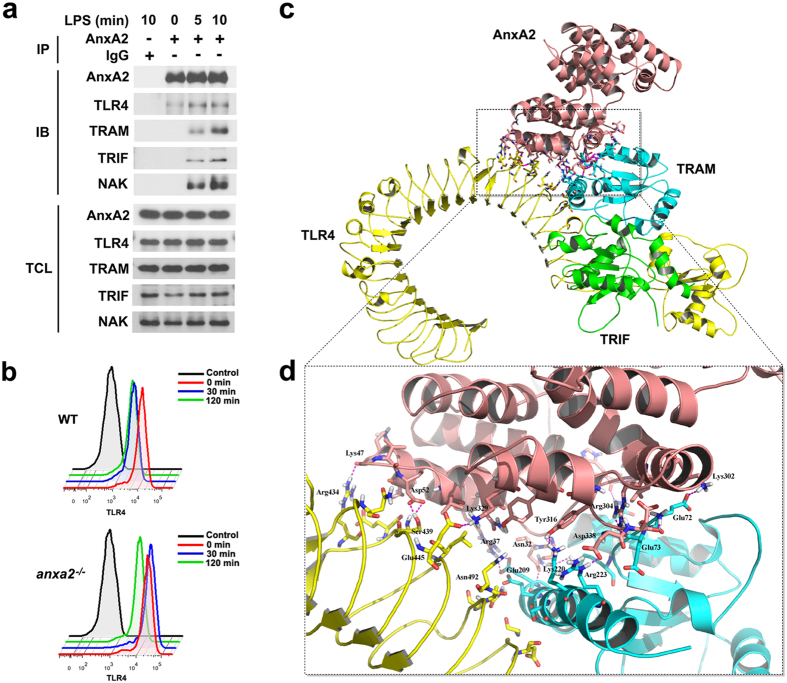
AnxA2 controls TLR4 internalization in peritoneal macrophages. (**a**) Immunoassay of lysates of peritoneal macrophages stimulated for 0, 5 or 10 min with LPS, then immunoprecipitated with IgG or AnxA2 antibody, assessed with anti-TLR4, anti-TRAM, anti-TRIF or anti-NAK. Total cell lysates (TCL) were applied for immunoblotting analysis as a control. Cropped blots are from gels run under the same experimental condition, and the full-length blots are presented in Supplementary Fig. 9e. (**b**) WT and *anxa2*^*−/−*^ peritoneal macrophages were stimulated with LPS (100 ng/ml), and TLR4 expression in cell membrane was analyzed by flow cytometry at 0, 30, 120 min after stimulation. (**c**) Structure of the quaternary complex model of AnxA2-TLR4-TRAM-TRIF. Color coding: TLR4, yellow; TRAM, blue; TRIF, green; and AnxA2, pink. (**d**) Image showed relatively clear interaction region between AnxA2 and TLR4 complex. Lys329 of AnxA2 formed electrostatic interactions with residue Glu445 of TLR4. Meanwhile, Arg37, Lys302, Arg304, Asp338 of AnxA2 was modelled to interact with Glu209, Glu72, Glu73, and Arg223 of TRAM via intensive electrostatic interactions, respectively. Lys47 and Asp52 of AnxA2 N-terminal formed hydrogen-bonding interactions with residues Arg434, Ser439 of TLR4. Besides, AnxA2 might form hydrophobic interactions with TLR4 complex. Only side chains of residues were shown for clarity.

**Figure 5:**
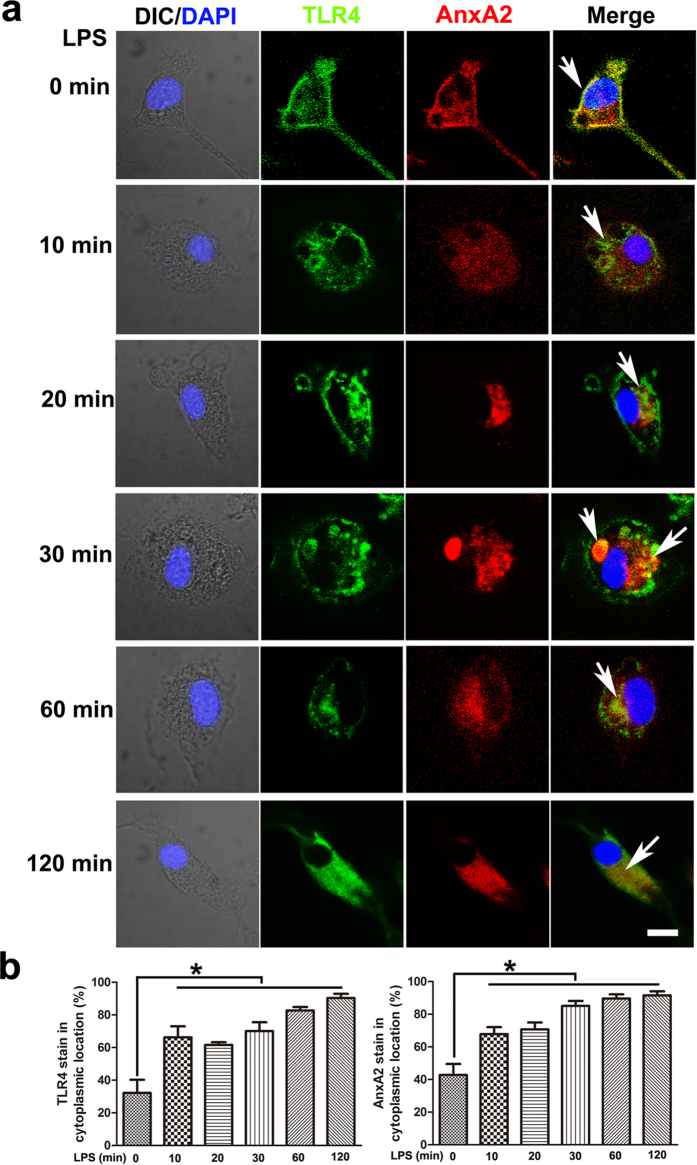
AnxA2 directly interacts with the LPS-activated TLR4 complex in peritoneal macrophages. CLSM microscopy of WT peritoneal macrophages activated for 0–120 min with 100 ng/ml LPS, and then stained with TLR4 and AnxA2 antibody, followed by corresponding fluorescent secondary antibodies. White arrows indicated AnxA2 dissociation from the plasma membrane ruffles, and showed substantially enhanced co-localization with TLR4 signalosome complex. DIC, differential interference contrast. Scale bars, 5 μm. Columns, mean; bars, SD (n = 10; ANOVA; *P < 0.05).

Although MH-S cells retain many properties of macrophages, including typical morphology, phagocytosis, esterase activity, and reactivity to LPS, these cells may somewhat lack the biological complexity of primary macrophages. To further elaborate the detail of TLR4-triggered inflammatory responses in macrophages, we used primary macrophages from mice isolated by peritoneal lavage (Supplementary Fig. 6). Immunoprecipitation with AnxA2 antibody showed that AnxA2 was recruited to the LPS-induced signalosome complex containing TLR4, TRAM and TRIF in WT peritoneal macrophages (Fig. 4a). Endocytosis of the TLR4 was significantly inhibited in peritoneal macrophages from *anxa2*^*−/−*^ mice relative to that in cells from WT mice (Fig. 4b). To explore a possible binding mode of AnxA2 and TLR4 complex, we performed computational studies using the ZDOCK program, a well-known protein-protein docking analysis program. The result indicated that AnxA2 may form stable complexes with TLR4 and TRAM via intensive electrostatic effect, hydrogen bonding, and hydrophobic interactions. Additionally, ZRank score was –103.849 (Fig. 4c,d). We also investigated whether AnxA2 influenced the localization of TLR4 under LPS stimulation. After 20 min stimulation with LPS in WT peritoneal macrophages, a large proportion of TLR4 was dissociated from the plasma membrane ruffles, followed by increased TLR4 staining in cytoplasm within 1 h. Meanwhile, AnxA2 aggregated as clusters along the cytoplasmic side of the membrane and showed substantially enhanced co-localization with TLR4 signalosome complex (Fig. 5). This biochemical evidence suggests a direct molecular interaction between AnxA2 and TLR4.

### Macrophage phagocytosis results in AnxA2 and TLR4 complex recruitment to the early endosome but not late endosome

**Figure 6:**
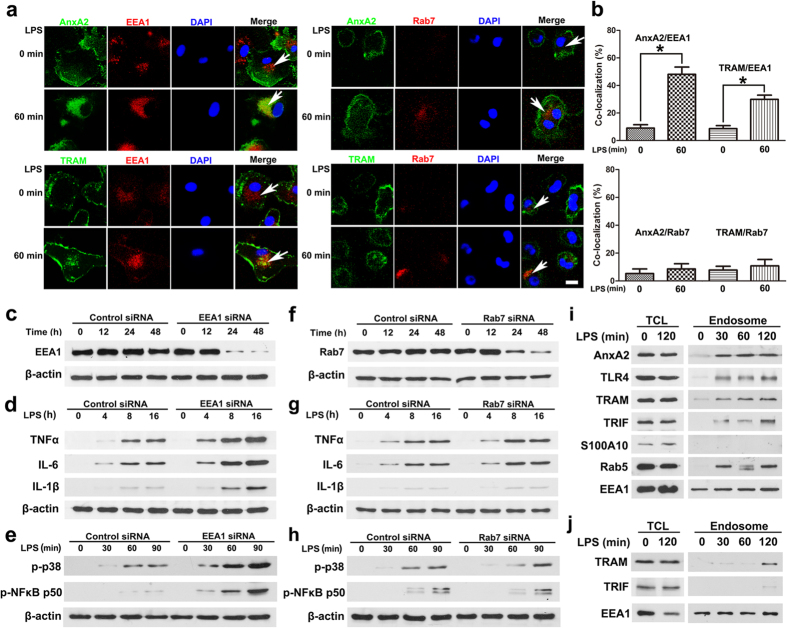
LPS treatment results in AnxA2 and TRAM recruitment to the early endosome but not late endosome in WT peritoneal macrophages. (**a**,**b**) Isolated macrophages were treated with 100 ng/ml LPS for 0–60 min, then stained with EEA1, AnxA2 (upper panels), or TRAM (lower panels) antibody. Meanwhile, isolated macrophages were treated with 100 ng/ml LPS for 0–60 min, and then stained with Rab7, AnxA2 (upper panels), or TRAM (lower panels) antibody. Images were visualized by CLSM. White arrows indicated AnxA2 and TRAM exhibited substantial co-localization with EEA1, but not Rab7 in LPS-treated macrophages. Scale bars, 5 μm. Columns, mean; bars, SD (n = 10; ANOVA; *P < 0.05). (**c**) Immunoblotting analysis of EEA1 in peritoneal macrophages after transfection with control siRNA or siRNA specific for EEA1. (**d**,**e**) Immunoblotting analysis of inflammatory cytokines and phosphorylated molecules in lysates of EEA1-silenced macrophages stimulated with 100 ng/ml LPS. (**f**) Immunoblotting analysis of Rab7 in peritoneal macrophages after transfection with control siRNA or siRNA specific for Rab7. (g,**h**) Immunoblotting analysis of inflammatory cytokines and phosphorylated molecules in lysates of Rab7-silenced macrophages stimulated with LPS. (**i**,**j**) Peritoneal macrophages were stimulated for 0, 30, 60 or 120 min with 100 ng/ml LPS, and endosomes were isolated using Dynabeads for immunoblotting analysis. AnxA2 and TLR4 signalosome complex were recruited in early endosomal localization of LPS-treated WT peritoneal macrophages (**i**); However, TLR4 signalosome complex were not recruited in early endosome of LPS-treated *anxa2*^*−/−*^ peritoneal macrophages (**j**). TCL, total cell lysates. Cropped blots are from gels run under the same experimental condition, and the full-length blots are presented in Supplementary Fig. 9f–i.

**Figure 7:**
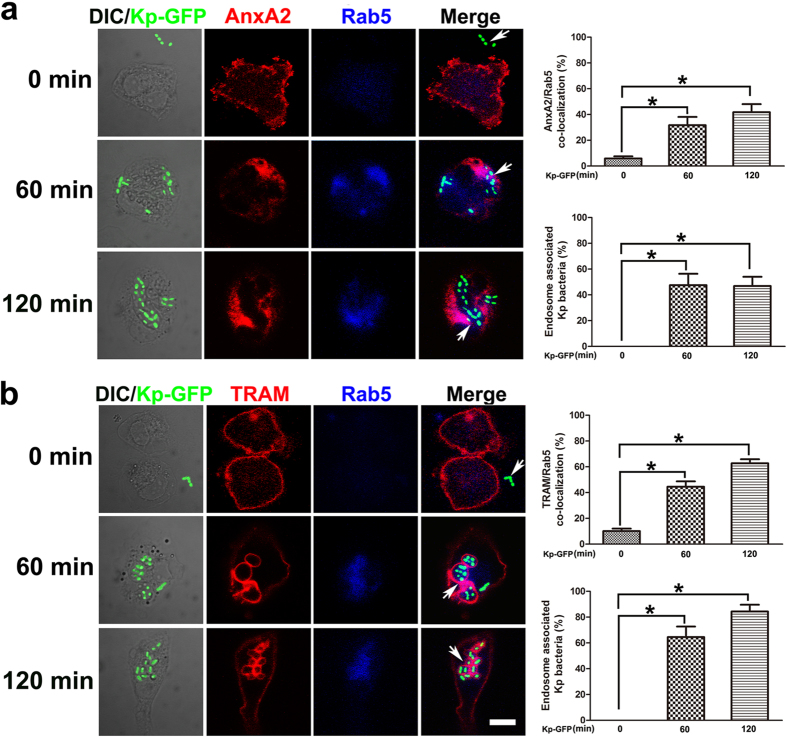
Bacterial phagocytosis induces AnxA2 and TRAM recruitment to endosome in peritoneal macrophages. WT peritoneal macrophages were treated with Kp-GFP for 2 h and analyzed by immunofluorescence assay. (**a**) Peritoneal macrophages were infected by Kp-GFP at an MOI (multiplicity of infection) of 10:1, and stained with Rab5 (a marker of early endosome) and AnxA2. (**b**) The other group of peritoneal macrophages with the same treatment was stained with Rab5 and TRAM antibodies. White arrows indicated the whole bacterial phagocytosis process. Scale bars, 5 μm. Columns, mean; bars, SD (n = 10; ANOVA; *P < 0.05).

To clarify the subcellular localization of TLR4 signalosome complex, we took advantage of double labeling immunofluorescence analysis to compare the intracellular distribution of TLR4 complex with various organelle markers in primary peritoneal macrophages. We found that AnxA2 and TRAM exhibited substantial co-localization with EEA1 (a marker of early endosome) in LPS-treated macrophages. In contrast, there was little co-localization of TLR4 complex with Rab7 (a marker of late endosome) in cells after LPS treatment (Fig. 6a, b). Meanwhile, we observed increased inflammatory cytokines and phosphorylated signaling molecules in LPS-stimulated EEA1-silenced macrophages in a time dependent manner, compared to those in LPS-stimulated Rab7-silenced macrophages (Fig. 6c–h). To further determine the TLR4 signalosome complex recruitment to endosome in LPS-treated macrophages, early endosomes were isolated from LPS-treated peritoneal macrophages and subjected to immunoblotting analysis. Our results demonstrated a substantial increase of AnxA2 protein in the isolated organelles in LPS-treated WT cells. Meanwhile, recruitment of TRAM and TRIF to the signalosome complex was also increased in LPS-treated WT cells, compared to that in *anxa2*^*−/−*^ peritoneal macrophages (Fig. 6i, j). In S100A10 knock-down peritoneal macrophages, early endosomes were isolated from LPS-treated macrophages, and the results of immunoblotting analysis indicated that TLR4 signalosome complex trafficking was S100A10 independent (Supplementary Fig. 7). CLSM microscopy also demonstrated greatly increased endosomal compartments, with concomitant recruitment of AnxA2 and TRAM in macrophages after Kp-GFP infection (Fig. 7). Taken together, these results indicated that early endosomal compartments in stimulated macrophages have recruited and contained TLR4 signalosome complex.

### AnxA2 confers protection from pro-inflammatory effects by promoting anti-inflammatory signaling

**Figure 8:**
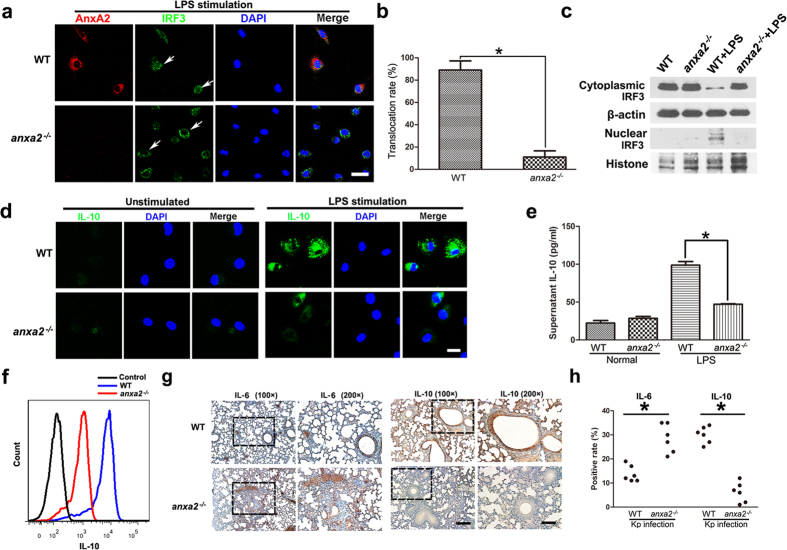
AnxA2 is required for optimal IL-10 production through IRF3. (**a**–**c**) CLSM microscopy of WT and *anxa2*^*−/−*^ peritoneal macrophages stimulated with 100 ng/ml LPS for 2 h, stained with AnxA2, IRF3 antibodies, and DAPI simultaneously. White arrows indicated IRF3 nuclear location in WT peritoneal macrophages, and IRF3 cytoplasmic location in *anxa2*^*−/−*^ peritoneal macrophages. Scale bars, 30 μm. These data were representative of three experiments. Columns, mean; bars, SD (n = 3; ANOVA; *P < 0.05). Meanwhile, WT and *anxa2*^*−/−*^ peritoneal macrophages were stimulated with LPS for 2 h, and IRF3 was detected by immunoblotting analysis in cytoplasmic and nuclear fractions in each group. Cropped blots are from gels run under the same experimental condition, and the full-length blots are presented in Supplementary Fig. 9j. (**d**–**f**) CLSM microscopy of WT and *anxa2*^*−/−*^ peritoneal macrophages stimulated with 100 ng/ml LPS for 12 h, then stained with IL-10. Scale bars, 10 μm. Quantitative assessment was further done by Elisa and flow cytometry to determine the exact amount of IL-10. Columns, mean; bars, SD (n = 3; ANOVA; *P < 0.05). (**g**,**h**) Evaluation of IL-6 or IL-10 by immunohistochemistry in lung tissue from Kp-treated WT and *anxa2*^*−/−*^ mice. Scale bars in left panels, 100 μm; in right panels, 50 μm. For each animal group, the positive area was counted in 6 randomly captured images (n = 6; ANOVA; *P < 0.05).

To delve into the detailed mechanism of AnxA2 effect on inflammation, we evaluated the pro-inflammatory and anti-inflammatory cytokine levels in primary peritoneal macrophages isolated from mice. Endosomal TRAM-TRIF signaling activated IRF3 and up-regulated anti-inflammatory cytokines. Translocation of IRF3 to the nucleus, which was required for its transcription activity, was much lower in *anxa2*^*−/−*^ macrophages than that in WT cells (Fig. 8a–c). In line with diminished IRF3 activation, *anxa2*^*−/−*^ macrophages expressed lower levels of IL-10 (Fig. 8d–f) and increased levels of pro-inflammatory cytokines (Supplementary Fig. 8) after stimulated with LPS for 12 h. To reflect the physiological relevance *in vivo*, we detected IL-10 and IL-6 levels in lung tissue from Kp-infected WT and *anxa2*^*−/−*^ mice using immunohistochemical staining. Lung tissue of *anxa2*^*−/−*^ mice exhibited much less IL-10 and more IL-6 than that in lung tissues of WT mice (Fig. 8g, h). These data suggested that depletion of AnxA2 down-regulated the production of IL-10, which was a possible cause leading to overzealous pro-inflammatory response.

## Discussion

Compartmentalization of TLR4 signaling has attracted much attention in recent years. Despite its importance, the precise nature of the compartment where intracellular TLR4 meets and interacts with internalized ligands remains poorly defined^16^,^17^. Here we have found that AnxA2 expression is a key step in negative regulation of inflammatory responses to bacterial infections, acting as a molecule to induce internalization of TLR4, followed by recruitment to early endosomal membranes. This process positively influences IL-10 production while negatively regulating NF-κB activity, presumably to control LPS response that can be lethal to the host.

Annexin family proteins, which can bind membranes via Ca^2+^ ion and negatively charged phospholipids, are considered scaffolding proteins to participate in membrane dynamics. AnxA2, a well characterized member of this family, is currently projected to play a key role in diverse processes range from cell migration to cell differentiation^18^,^19^. AnxA2 has been described as a surface binding receptor for a number of different molecules, and predominantly acts as an anti-inflammatory agent largely because of its structural similarities to annexin A1 (AnxA1), which exhibits anti-inflammatory activities in several animal models of inflammation^20^,^21^,^22^. It has also been demonstrated that AnxA2 plays a crucial role in the clearance of apoptotic lymphocytes^23^. Recently, a series of studies on plasma membrane damage have indicated that AnxA2 mediates repair mechanisms in dysfunctional cytoplasma membranes^24^,^25^. Here we have provided evidence that AnxA2 may be a negative regulator of bacteria-triggered inflammatory responses. After intranasal challenge with Kp bacteria, 50% of the *anxa2*^*−/−*^ mice died within 30 h, whereas all WT mice survived up to 50 h. The higher mortality in *anxa2*^*−/−*^ mice correlated with more serum pro-inflammatory cytokines including TNFα, IL-6 and IL-1β, which were attributed to the lethal pathology of endotoxin shock. Meanwhile, we found similar results in another experiment using bacterial peritonitis model. However, AnxA2 protein lacks transmembrane domain, it remains speculative on its signaling and subsequent effects on bacterial infection^26^.

TLRs recognize a wide range of highly conserved microbial ligands including bacterial cell wall components, bacterial lipoproteins, and bacterial nucleic acids, which is linked to a cascade of events that activate innate immune responses and priming of adaptive immune responses. LPS is a principal component of Gram-negative bacterial membrane and elicits inflammatory responses that may lead to shock and ultimately death. TLR4 is expressed on antigen-presenting cells including macrophages and dendritic cells, and indispensable for LPS responses^27^,^28^,^29^,^30^. To investigate whether AnxA2 interacted with the TLR signaling pathways in macrophages, we examined the activation kinetics of the MAPK and NF-κB pathways, which are downstream of TLR4 signaling. In MH-S alveolar macrophages with depletion of AnxA2 by siRNA, activation of MAPK and NF-κB pathways was enhanced during LPS stimulation. Although MH-S retains many of the properties of alveolar macrophages, this cell line may not represent the biological complexity of primary cells isolated from mammals with normal physiological conditions. To further confirm the inflammatory effects of macrophages, we used primary peritoneal macrophages in the following study.

In a recent investigation, TLR4 is purified from endothelial cells using AnxA2 immobilized columns^31^. Moreover, β_2_ glycoprotein I (β_2_-GPI) and TLR4 interactions have been demonstrated in monocytes in which AnxA2 is richly expressed^32^. Finally, a signaling complex on the surface of endothelial cells consists of AnxA2, TLR4, calreticulin and nucleolin, which mediates cell activation by anti-β_2_-GPI antibodies^33^. However, the precise roles of AnxA2 in the TLR4-mediated pathogen recognition remain unknown. Here we used primary peritoneal macrophages in which AnxA2 was genetically inactivated. We found that LPS-mediated endocytosis of TLR4 was significantly inhibited in macrophages from *anxa2*^*−/−*^ mice relative to that in cells from WT mice. Further immunoprecipitation showed that AnxA2 was recruited to the LPS-induced signalosome complex which contained TLR4, TRAM, TRIF, and NAK in WT macrophages. It has also remained unclear whether this complex plays a positive or negative role in TLR4 signaling, with published evidence supporting both possibilities^1^,^34^.

TLR4 induces two independent signaling pathways that are regulated by the TIRAP-MyD88 and TRAM-TRIF adaptors, respectively. The TIRAP-MyD88-dependent pathway induces rapid activation of serine-threonine kinases which trigger pro-inflammatory signal transduction, whereas TLR4 internalization promotes TRAM-TRIF signaling in endosomes and induces IFN-β, IL-10, and other anti-inflammatory cytokines^14^,^35^,^36^,^37^,^38^. Here we found that AnxA2 was involved in the TRAM-TRIF-dependent signaling pathway in the early endosomal location, after LPS induced endocytosis of TLR4. At least three independent experiments supported this conclusion. First, after stimulation with LPS, TLR4 in WT peritoneal macrophages dissociated from the plasma membrane ruffles, and showed substantially enhanced co-localization with aggregated AnxA2 clusters in cytoplasmic side of the membrane. Secondly, immunoblotting and immunofluorescence analysis demonstrated well-defined EEA1 positive endosomal compartments and the endosomal distribution of TLR4 complex. Thirdly, genetically inactivated AnxA2 disrupted the TRAM-TRIF-dependent translocation of IRF3, consequently inhibited IL-10 expression. However, several reports have indicated AnxA2 endosomal binding is associated with increased S100A10 recruitment, and the heterotetrameric complex (AnxA2-S100A10) serves in the pro-inflammatory process^39^,^40^. Our results argue against this mechanism in the bacterial infection process, because S100A10 was not detected in isolated endosomes from LPS-treated macrophages.

In summary, our results have demonstrated that AnxA2 molecules interact with a TRAM-TRIF adaptor complex and subsequently regulate TLR4 endosomal signaling and its anti-inflammatory action during endotoxemia. Our findings provide new insight into the negative regulation of TLR signaling and indicate a previously unidentified function of AnxA2 molecule in the innate inflammatory responses.

## Methods

### Bacteria preparation and pulmonary infection

*Klebsiella pneumoniae* (Kp, ATCC43816) was kindly provided by Dr. V. Miller (University of North Carolina, Chapel Hill, NC)^41^. Bacteria were grown for 14 h in LB broth at 37 °C with shaking. The bacteria were pelleted by centrifugation at 5000 *g*. C57BL/6 female mice (6–8 weeks) were obtained from the Jackson Laboratory (Bar Harbor, ME). *anxa2*^*−/−*^ mice (C57BL/6 genetic background) were kindly provided by Dr. K. Hajjar (Cornell University, Ithaca, NY)^42^. To generate anxa2^*−/−*^ mice, exon 3 and 4 of *anxa2* were disrupted with a cassette containing neomycin phosphotransferase driven by the phosphoglucokinase promoter. *anxa2*^*−/−*^ mice were back-crossed 6 generations with C57BL/6 before experiments. Mice were anesthetized with 80 mg/kg ketamine, then intranasally instilled 1 × 10^5^ CFU of Kp in 50 μl of PBS. Mice were observed for symptoms and euthanized when they were moribund. Mice were kept and bred in the animal facility at the University of North Dakota, and all animal experiments were approved by the institutional animal care and use committee (UND IACUC) and executed under National Institutes of Health guidelines.

### Vascular permeability measurements in mouse lung tissue

Twenty four hours after intranasal infection, 100 μl of 0.5% Evans blue dye was injected into the caudal vein for 120 min as described previously^43^. After mice were sacrificed, lungs were perfused with PBS, and Evans blue was extracted from homogenized lung tissue by incubating in 70% acetone for 24 h at 55 °C. Evans blue was quantified by measuring the absorbance at 615 nm.

### Histological analysis

After infection in mice, lung or other tissues were fixed in 4% formaldehyde for 48 h and then processed to hematoxylin and eosin staining (H&E staining) in AML Laboratories (Baltimore, MD)^44^.

### TUNEL assay

Cell apoptosis in lung tissues was determined using the TUNEL assay following the manufacturer’s instructions (Promega, Madison, WI). The samples were counterstained with DAPI, and the number of TUNEL-positive cells was quantified by fluorescence microscopy^45^.

### Bacterial burden assay

24 h after infection, Lung, spleen, liver, and kidney tissues were homogenized and spread on LB plates. Meanwhile, BAL fluid and blood were spread directly on LB plates. The plates were cultured overnight and the colonies were counted^46^.

### Primary alveolar macrophages isolation

Primary alveolar macrophages were obtained from BAL fluid as previously described^47^,^48^. Briefly, the mouse lungs were lavaged three times with 1 ml normal saline containing 1% fetal bovine serum (Invitrogen, Carlsbad, CA). The BAL fluid was centrifuged and resuspended in RPMI 1640 medium supplemented with 10% fetal bovine serum. Cells were incubated overnight and then nonadherent cells were removed by washing with normal saline.

### NBT assay

Mice were killed at the indicated time after Kp infection and the alveolar macrophages were collected from BAL fluid. Primary alveolar macrophages were cultured in a 96-well plate (1 × 10^4^ cells per well). ROS production was detected by NBT assay by incubating the cells for 120 min in PBS containing 0.2% NBT. NBT was reduced by ROS to the dark blue formazan, which was dissolved in DMSO and its absorbance determined at 560 nm^49^.

### H_2_DCF assay

H_2_DCF dye emitted green fluorescence upon reaction with superoxide. Cells were treated as above and incubated in 10 μM H_2_DCF dye. Fluorescence was measured after 10 min incubation using the fluorimeter (BioTek, Winooski, Vermont)^44^.

### JC-1 assay

Mitochondrial function was detected using JC-1 probe, which produces green fluorescence in the cell cytoplasm and red fluorescence when it aggregates in respiring mitochondria^44^. Cells were treated as above and incubated in 5 μg/ml JC-1 solutions for 10 min at 37 °C. Cells were excited at 488 nm and JC-1 emission was collected by fluorimeter (BioTek).

### MTT assay

Cells were treated as above and incubated in 0.5 mg/ml MTT solutions for 3 h at 37 °C. 100 μl stop solution (10% SDS) was added per well and the plate was left overnight. The absorbance was recorded using a 96-well spectrophotometer (Thermo Scientific, Waltham, MA) at a wavelength of 570 nm^44^.

### ELISA

24 h after infection, BAL fluid and serum of mice were collected and applied for inflammatory cytokine profiling assay. Cytokine concentrations were measured by ELISA kits following the manufacturer’s instructions (eBioscience, San Diego, CA)^49^.

### Mouse peritonitis model and luminescent imaging

Kp Xen-39, a bioluminescent pathogenic bacterium strain, was cultured and prepared as described previously^44^. Groups of mice were inoculated intraperitoneally with a lethal dose of Kp Xen-39 (2 × 10^5^ CFU/mouse). At hourly intervals following inoculation, groups of mice were anesthetized with an intraperitoneal injection of 80 mg/kg ketamine. The anesthetized mice were transferred to the imaging chamber, ventral side up, and imaged with Xenogen IVIS optical imaging system (Caliper Life Sciences, Hopkinton, MA). The animal experiments were approved by the institutional animal care and use committee (UND IACUC) and executed under National Institutes of Health guidelines.

### Permeability measurements in peritoneum

24 h after intraperitoneal infection, 100 μl of 0.5% Evans blue dye was injected into the caudal vein for 120 min. After mice were sacrificed, peritoneal cavity was washed with 5 ml of ice-cold PBS, and Evans blue was quantified as an indicator of peritoneal permeability by measuring the absorbance at 615 nm^50^.

### Cell transfection with siRNA

Murine MH-S alveolar macrophages were obtained from American Type Culture Collection (ATCC, Manassas, VA) and cultured following the manufacturer’s instructions. In a 6 well culture plate, 5 × 10^5^ MH-S cells per well were seeded in 2 ml antibiotic-free medium and grown overnight. Transient transfections were performed using 20 pmol siRNA (Santa Cruz Biotechnology, Santa Cruz, CA) and 5 μl Lipofectamine 2000 reagent (Life Technologies, Rockville, MD)^47^.

### Expression profiling with a Primer PCR Array

Total RNA was isolated from MH-S cells with TRIzol reagent (Life Technologies) and reverse transcribed. The cDNA was stored at 4 °C until hybridization to a microarray including 84 key genes in response to innate and adaptive immunity (Bio-Rad, Hercules, CA). Briefly, an initial incubation of 5 min at 95 °C was performed, followed by 40 cycles consisting of template denaturation (15 s at 95 °C) and one-step annealing and elongation (30 s at 55 °C), with the C1000 Touch real-time PCR system. Data were extracted and analyzed with PrimePCR Analysis Application 1.0 software (Bio-Rad)^51^.

### Western blot analysis

Cells were lysed with buffer containing 1% NP-40 and proteinase inhibitor cocktail (Thermo Scientific, Rockford, IL). Protein concentrations were determined by Bradford assay (Bio-Rad) and equalized before loading. Cellular protein from each sample was applied to 8% to 12% SDS-PAGE gels and probed with specific antibodies including EEA1, Rab7, RIP1 (Cell Signaling Technology, Danvers, MA), AnxA2, p-IκBα, p-IKKα, p-ERK, p-p38, p-JNK, p-NFκB p65, p-NFκB p50, TNFα, IL-6, IL-1β, and β-actin (Santa Cruz Biotechnology). Blots were developed with horseradish peroxidase-conjugated secondary antibodies (Santa Cruz Biotechnology) and chemiluminescent substrate on Fuji X-ray films^52^.

### Luciferase assay

AnxA2 siRNA transfection was performed as described previously. AnxA2 knock-down or normal MH-S cells were transiently transfected with pNF-κB-luc plasmid (Promega) for 24 h, then stimulated with 100 ng/ml LPS (Sigma-Aldrich, St. Louis, MO) in RPMI 1640 medium for another 12 h. Cell lysates were subjected to luciferase activity analysis using the Dual-Luciferase Reporter Assay System (Promega) following the manufacturer’s instruction^47^. Firefly luciferase and renilla luciferase activity were also measured by Xenogen IVIS optical imaging system (Caliper Life Sciences).

### Primary peritoneal macrophages isolation

Murine resident peritoneal macrophages were obtained by peritoneal lavage with 5 ml RPMI 1640 containing 10% FBS. Cells were incubated overnight and then washed with normal saline to eliminate nonadherent cells^53^. Purity of the cells was determined by F4/80 and CD11b flow cytometry analysis.

### Immunoprecipitation

The primary peritoneal macrophages were stimulated with LPS, then lysed with lysis buffer supplemented with protease inhibitor (Thermo Scientific). 1 ml cell lysate was immunoprecipitated with 2 μg AnxA2 antibody and 10 μl protein A sepharose beads. Immunoblotting assays were done as described previously^54^.

### Computational analysis

The AnxA2-TLR4-TRAM-TRIF complex model was constructed using the ZDOCK program. Briefly, 3D structures of AnxA2 (PDB code: 4HRE) and TLR4 (PDB code: 3VQ1) were from Protein Data Bank (PDB) database, while 3D structures of TRAM and TRIF were constructed using SWISS-MODE, which is a fully automated protein structure homology-modelling server. TRAM was blindly docked onto TRIF using the ZDOCK program implemented in Discovery Studio 3.1. The TRAM-TRIF complex model with the highest ZRank score served as the template for constructing the complex model of TLR4-TRAM-TRIF. Similarly, AnxA2 was docked onto the existing TLR4-TRAM-TRIF model to generate the final AnxA2-TLR4-TRAM-TRIF quaternary complex model. The protein-protein interactions were visualized using the PyMOL program^55^.

### Immunofluorescence assay

MH-S cells and primary peritoneal macrophages were fixed by 2% formaldehyde and permeabilized in 0.5% Triton X-100, then blocked in 1% BSA for 30 min. The indicated primary antibody was incubated with the cells overnight at 4 °C. After incubation with appropriate fluorescence-conjugated secondary antibody (Life Technologies) for 30 min, the images were captured by LSM 510 Meta confocal microscope (Carl Zeiss, Thornwood, NY)^56^.

### Flow cytometry analysis

Primary peritoneal macrophages were harvested and applied for the experiments. Indicated antibodies were added for 1 h, followed by incubation with appropriate fluorescence-conjugated secondary antibody (Life Technologies) for 30 min. Cells were washed and harvested for flow cytometry^57^.

### Endosomes isolation

Endosomes were isolated using Dynabeads (M-280 Sheep anti-Rabbit IgG) following the manufacturer’s instructions (Life Technologies). In brief, Dynabeads were incubated with EEA1 antibody for 1 h, and mixed with crude fraction of primary peritoneal macrophages. Endosomes bound to the Dynabeads were separated by the magnet^58^.

### Immunohistochemistry

The lung tissue sections were deparaffinized and subjected to microwave-mediated antigen retrieval in citrate buffer (pH = 6.0), prior to blocking with 1% BSA for 30 min at 25 °C. IL-10 or IL-6 antibody (Santa Cruz Biotechnology) was diluted 1:100 and incubated with the sections overnight at 4 °C. HRP polymer-conjugated goat anti-mouse secondary antibody and DAB (Abcam, Cambridge, MA) were applied to indicate the positive area^59^.

### Statistical analysis

Statistical analysis was done with SPSS 18.0 software. Data was performed by One-way ANOVA (Tukey post hoc test) for comparing two groups and log rank test for Kaplan-Meier survival curves. Differences were considered significant if *P* < 0.05.

## Additional Information

**How to cite this article**: Zhang, S. *et al.* Annexin A2 binds to endosomes and negatively regulates TLR4-triggered inflammatory responses via the TRAM-TRIF pathway. *Sci. Rep.* **5**, 15859; doi: 10.1038/srep15859 (2015).

## Supplementary Material

Supplementary Information
