# Supplementary material for: Annexin A2 binds to endosomes and negatively regulates TLR4-triggered inflammatory responses via the TRAM-TRIF pathway
Source: Sci Rep. 2015 Nov 3;5:15859. doi: 10.1038/srep15859 (PMC4630631; doi:10.1038/srep15859)
Supplement: Supplementary Information [file srep15859-s1.pdf]

Supplementary Information

Title of manuscript:

Annexin A2 binds to endosomes and negatively regulates TLR4-triggered inflammatory responses via the TRAM-TRIF pathway

Authors:

Shuang Zhang, Min Yu, Qiang Guo, Rongpeng Li, Guobo Li, Shirui Tan, Xuefeng Li, Yuquan Wei & Min Wu

Supplementary information includes:

Supplementary Figures S1-S9

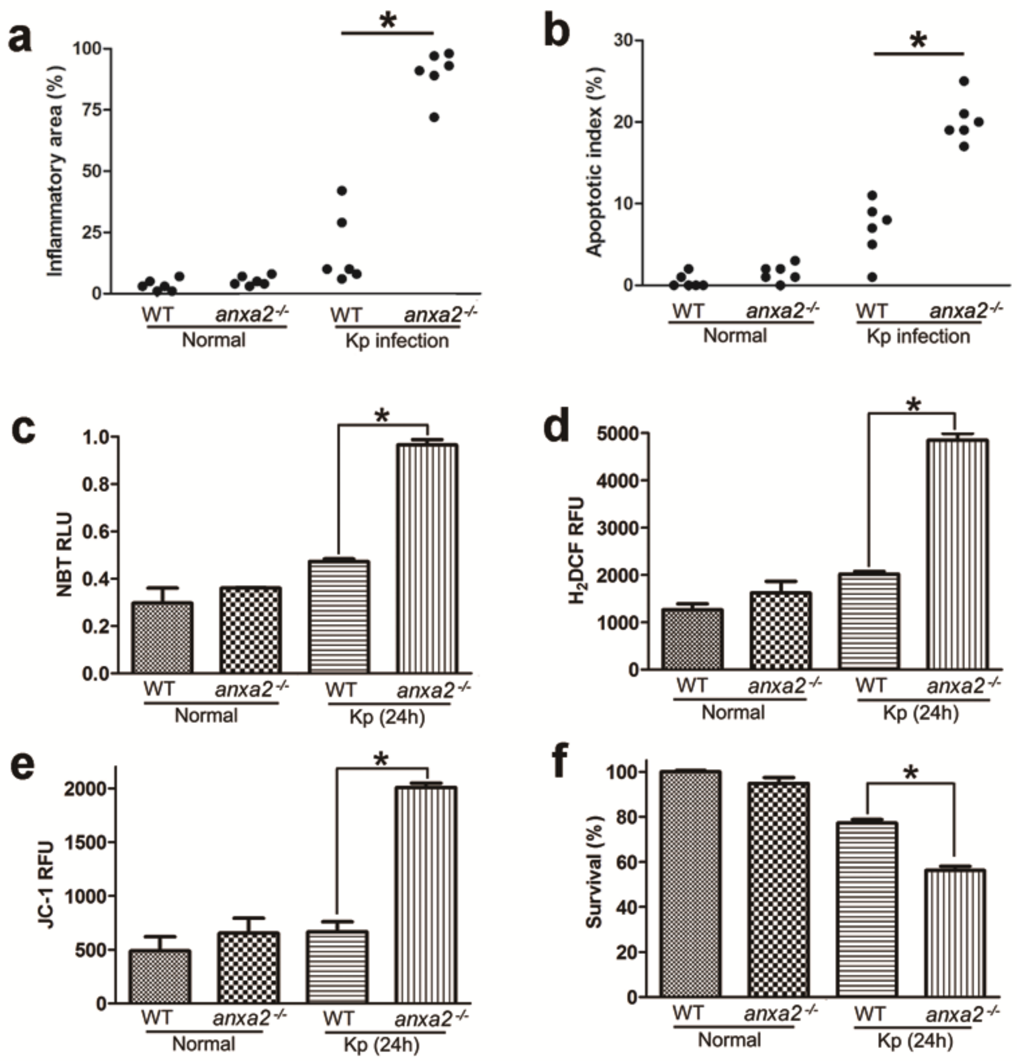

Supplementary figure S1. AnxA2 attenuates Kp-induced pulmonary inflammation. (a) The statistical data of Fig. 1c. Inflammatory area was calculated by dividing the thickened alveolar interstitium by the whole lung tissue. For each animal group, the inflammatory area was counted in 6 randomly captured images (n=6; ANOVA; *, $P<0.05$). (b) The statistical data of Fig. 1d. Apoptotic index was calculated by dividing the number of TUNEL-positive cells by the total number of cells. For each animal group, the apoptotic index was counted in 6 randomly captured images (n=6; ANOVA; *, $P<0.05$). (c) Alveolar macrophages were isolated from BAL fluid, and

superoxide production was detected using NBT assay. The absorbance was recorded using a 96-well spectrophotometer at a wavelength of 560 nm (n=3; ANOVA; *, P<0.05). (d) The NBT results were confirmed by H_2DCF assay. It was evaluated with the fluorimeter at an excitation wavelength of 488 nm and observation wavelengths of 525 nm (n=3; ANOVA; *, P<0.05). (e) Mitochondrial potential of alveolar macrophages was assessed by the JC-1 fluorescence assay, which provided conclusive evidence of cell apoptosis. The fluorescence was quantified at 530 nm by fluorimeter (n=3; ANOVA; *, P<0.05). (f) MTT assay determined the proliferation of the alveolar macrophages, and the absorbance was recorded using a 96-well spectrophotometer at a wavelength of 570 nm (n=3; ANOVA; *, P<0.05).

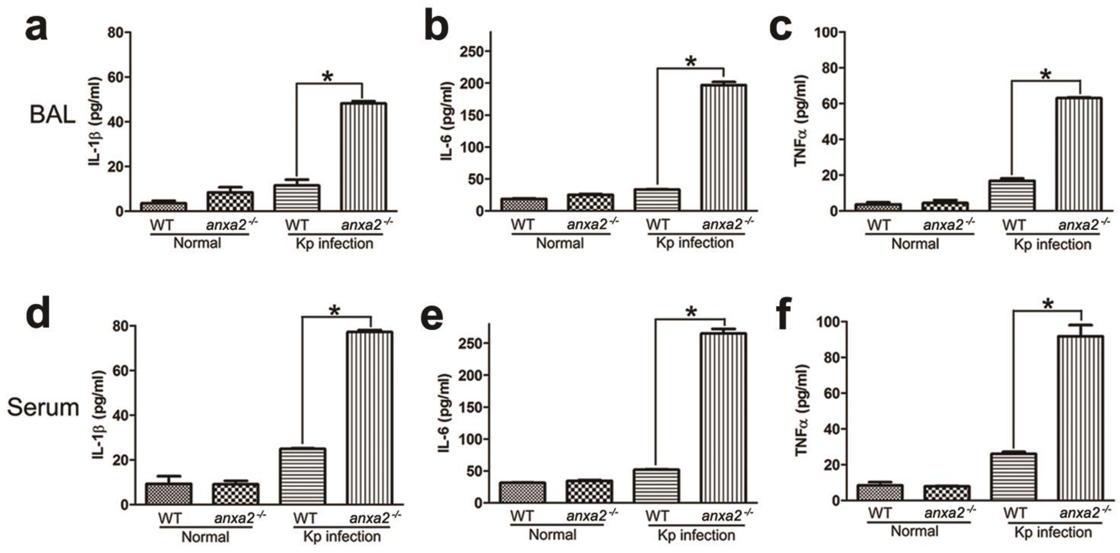

Supplementary figure S2. Pro-inflammatory cytokines are higher in local and systemic fluid circulation in the mice lacking AnxA2. 24 h after Kp infection, BAL fluid (a, b, c) and serum (d, e, f) were collected in each group, and

inflammatory cytokines (IL-1β, IL-6 and TNFα) were assessed by ELISA. Each assay was performed in three replicates (n=3; ANOVA; *, $P<0.05$).

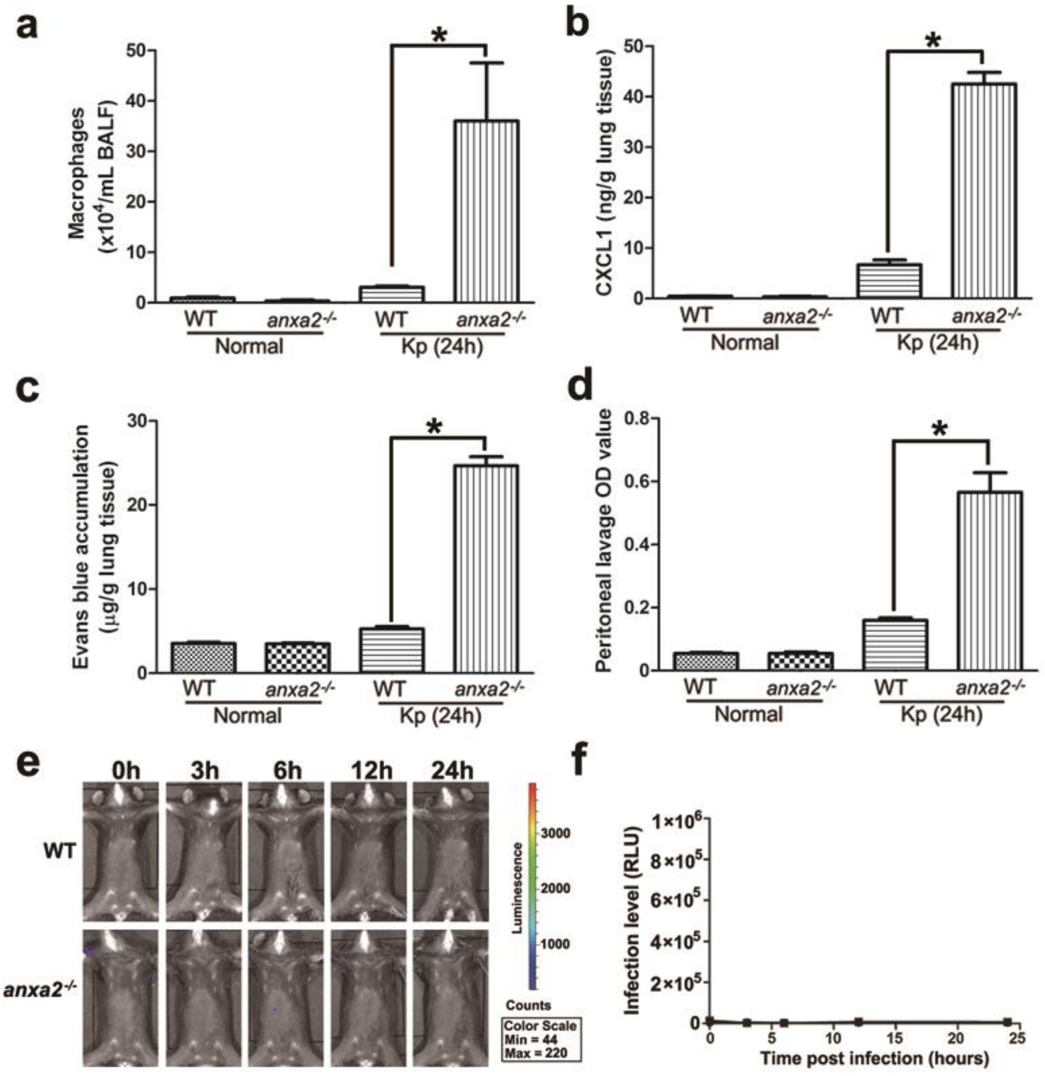

Supplementary figure S3. (a, b) AnxA2 deficiency promoted CXCL1 production and associated macrophage recruitment after Kp challenge (n=3; ANOVA; *, P<0.05). Lung interstitial macrophages and CXCL1 were determined 24 h after Kp infection in WT or *anxa2*$^{-/-}$ mice. (c, d) AnxA2 attenuated bacteria-induced vascular permeability (n=5; ANOVA; *, P<0.05). Vascular permeability in lung tissue or peritoneum was assessed by Evans blue accumulation as described

in the Methods. (e, f) WT or *anxa2*$^{-/-}$ mice were inoculated intraperitoneally with the heat-killed bioluminescent Kp (2×10^5 CFU/mouse), and whole animal imaging of bioluminescence was detected by IVIS XRII system at different time points (n=5; ANOVA; *, P<0.05).

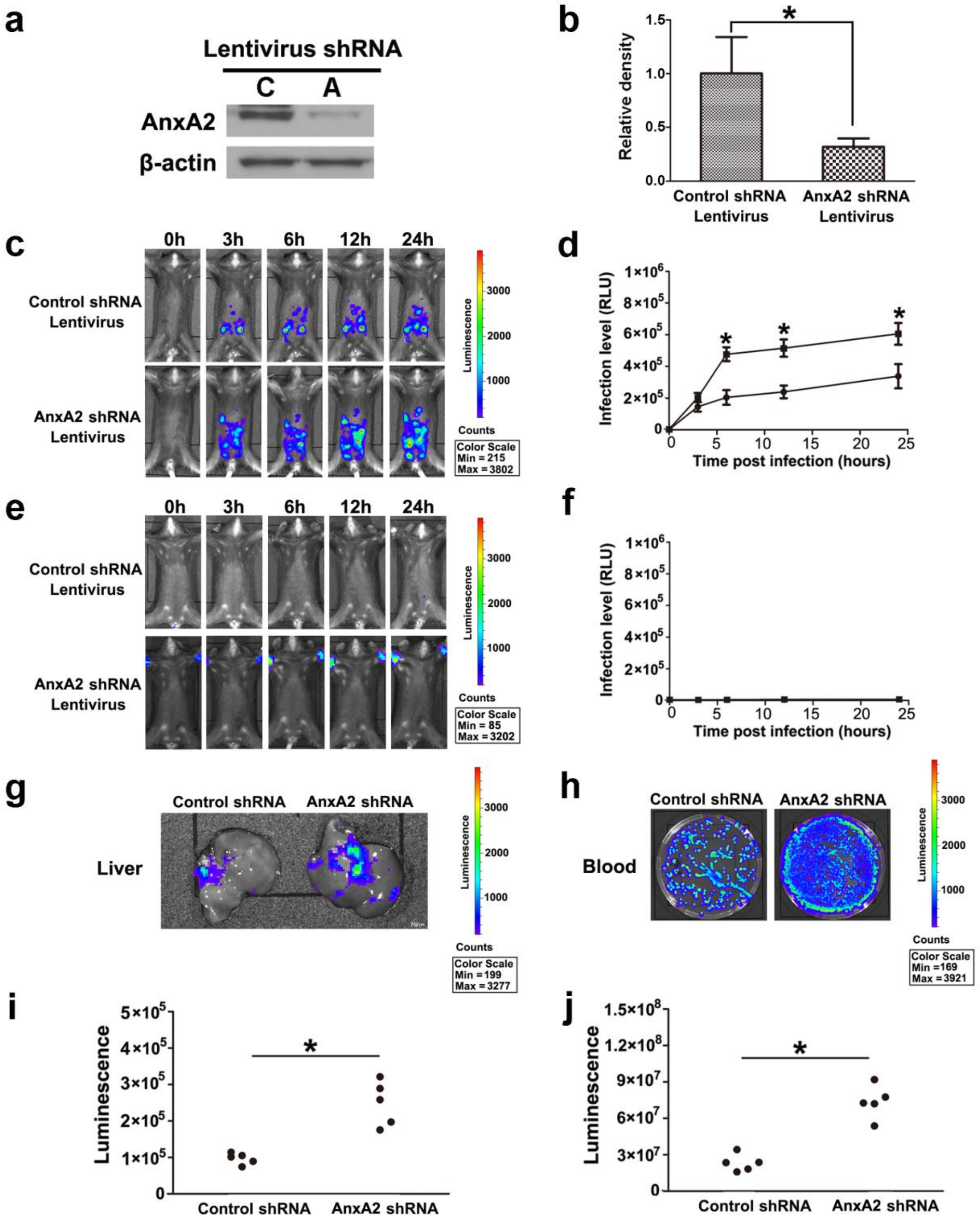

Supplementary figure S4. Decreased AnxA2 promotes Kp spread from

peritoneal cavity to bloodstream. (a, b) 2×10^5 AnxA2 shRNA lentiviral particles or control shRNA lentiviral particles was injected i.p. in WT mice, respectively. After 24 h, peritoneal macrophages were collected and lysed with buffer containing 1% NP-40, then applied for AnxA2 protein detection. Each assay was replicated three times, and the blots were quantified using densitometry with Quantity One (n=3; ANOVA; *, P<0.05). (c, d) 2×10^5 AnxA2 shRNA lentiviral particles was injected i.p. at 1 day before Kp infection. At indicated time points after infection, decreased AnxA2 promoted bacterial growth in peritoneal cavity of WT mice (n=5; ANOVA; *, P<0.05). (e, f) The heat-killed bioluminescent Kp was served as the control. (g, i) 24 h after infection, imaging of livers showed enhanced bacterial spread in WT mice with decreased AnxA2 (n=5; ANOVA; *, P<0.05). (h, j) 24 h after infection, blood bacterial plating was cultured overnight and demonstrated a higher systemic colonization in WT mice with decreased AnxA2 (n=5; ANOVA; *, P<0.05).

a

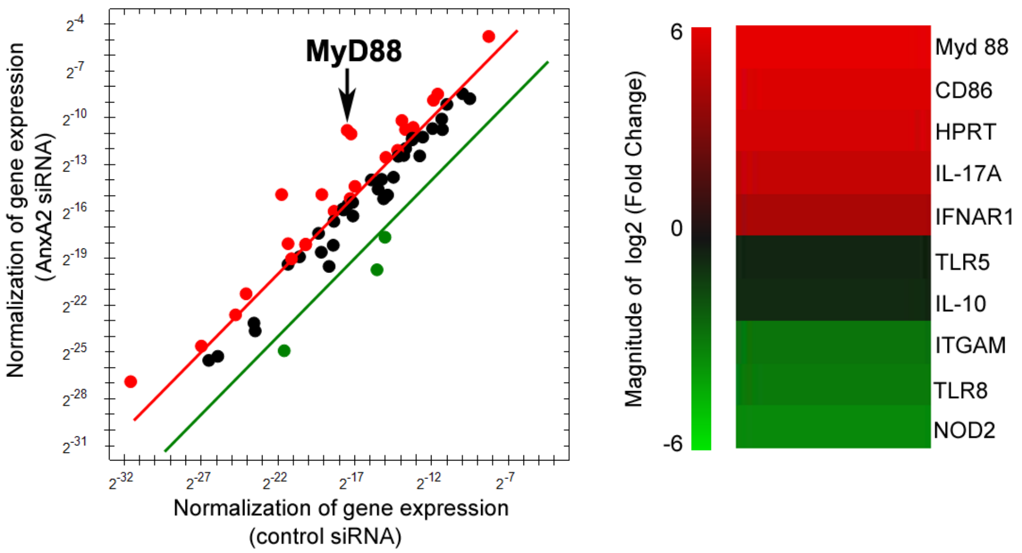

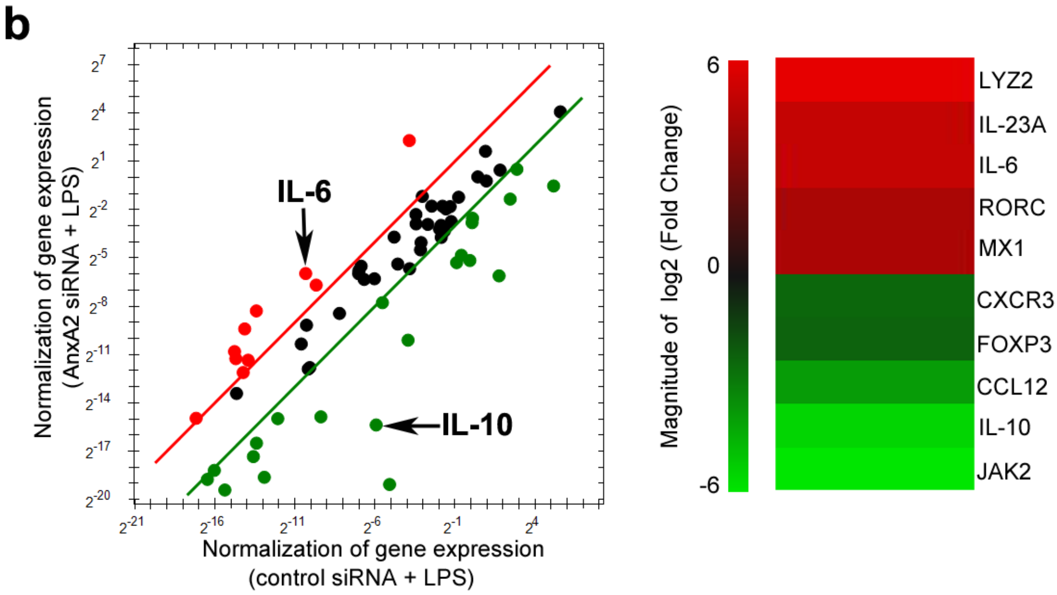

Supplementary figure S5. Microarray analysis of gene expression in MH-S cells upon stimulation with LPS. (a) MH-S cells were transfected with AnxA2 siRNA or control siRNA for 48 h. Genes with a greater than four-fold change ($P<0.05$) were depicted, and the heat map showed the top 10 most significantly changed genes. (b) MH-S cells were transfected with AnxA2 siRNA or control siRNA for 48 h, then stimulated with 100 ng/ml LPS for 1 h. Genes with a greater than four-fold change ($P<0.05$) were depicted, and the heat map showed the top 10 most significantly changed genes.

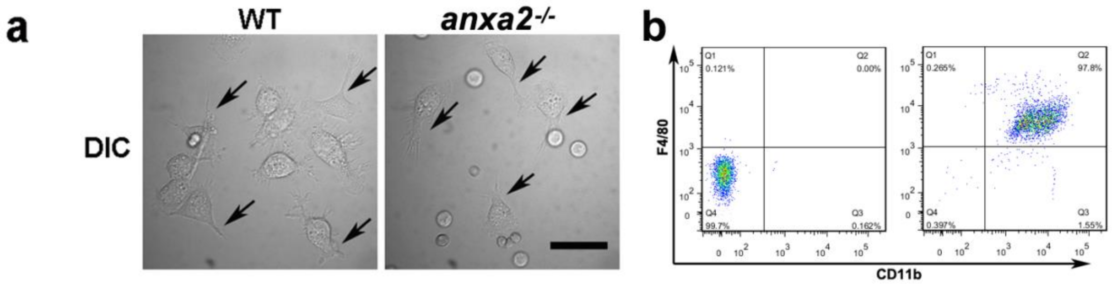

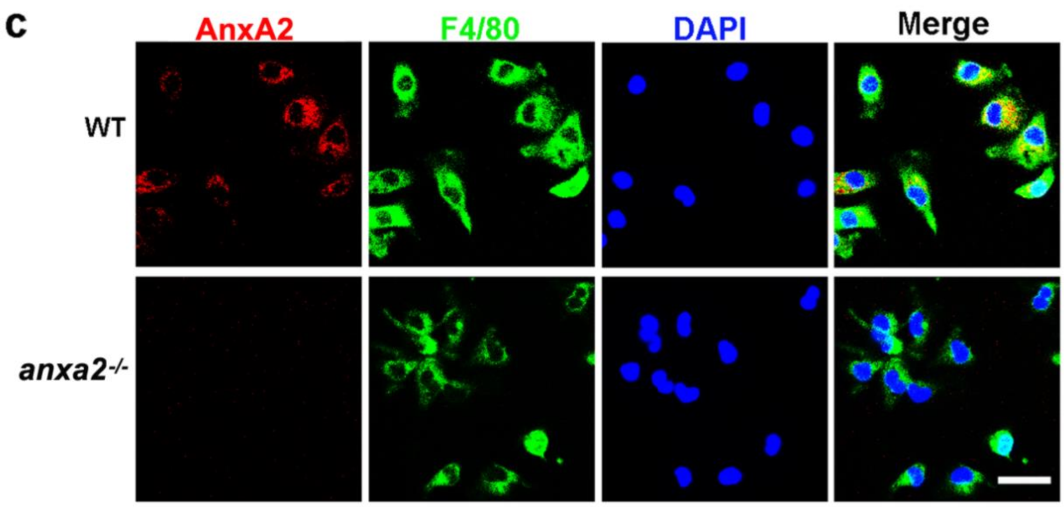

Supplementary figure S6. Isolated primary peritoneal macrophages were imaged and identified. (a) The cells retained the typical macrophage morphology, such as large cells with irregular pseudopodia (arrowhead). Scale bars, 30 μm. (b) Flow cytometric analysis was used to identify the primary peritoneal macrophages. F4/80 and CD11b, which were macrophage-specific markers, were found in 97.8% primary peritoneal cells. (c) Cells separated from WT and *anxa2*$^{-/-}$ mice were stained with F4/80 and AnxA2 by immunofluorescence. Scale bars, 30 μm.

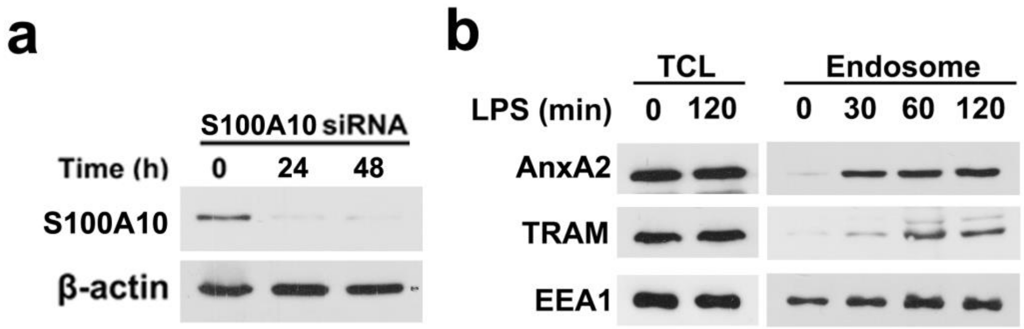

Supplementary figure S7. TLR4 signalosome complex trafficking is S100A10 independent. (a) Primary peritoneal macrophages were transfected with 20

pmol control or S100A10 siRNA for 0, 24, 48 h, and the S100A10 expression was detected by immunoblotting analysis. (b) S100A10 knock-down or normal peritoneal macrophages were stimulated for 0, 30, 60 or 120 min with 100 ng/ml LPS, and endosomes were isolated using Dynabeads for immunoblotting analysis.

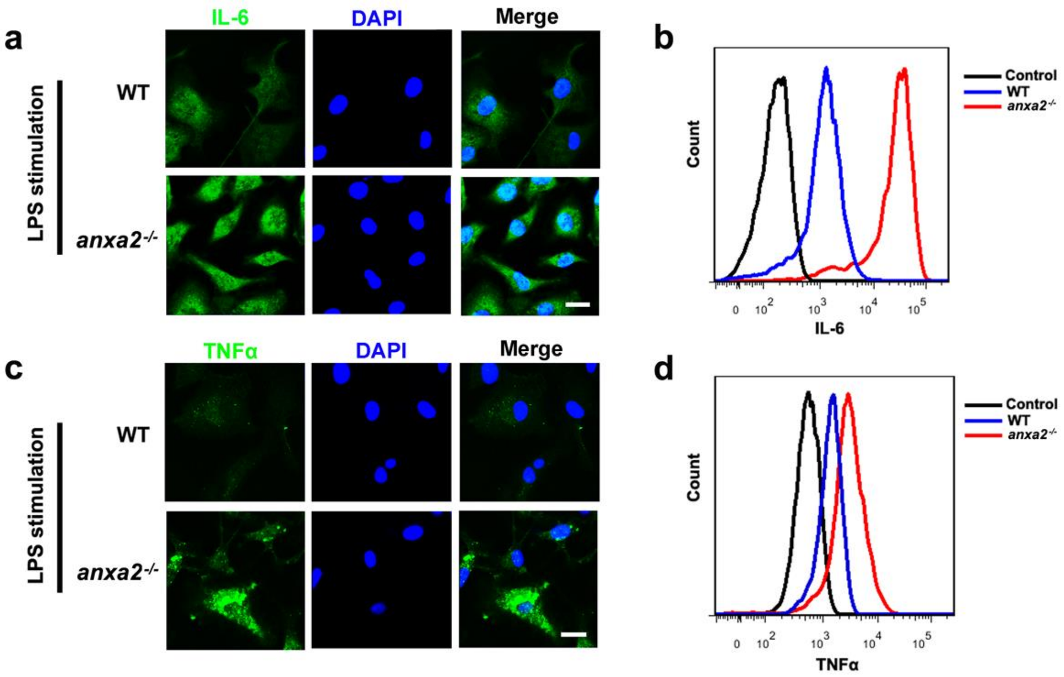

Supplementary figure S8. *Anxa2*$^{-/-}$ peritoneal macrophages augments endotoxin-mediated pro-inflammatory cytokines. (a, b) WT and *anxa2*$^{-/-}$ peritoneal macrophages were stimulated with 100 ng/ml LPS for 12 h, then stained with IL-6. Scale bars, 10 μm. Quantitative assessment was further done by flow cytometry. (c, d) WT and *anxa2*$^{-/-}$ peritoneal macrophages were stimulated with 100 ng/ml LPS for 12 h, then stained with TNFα. Scale bars, 10 μm. Quantitative assessment was further done by flow cytometry.

a

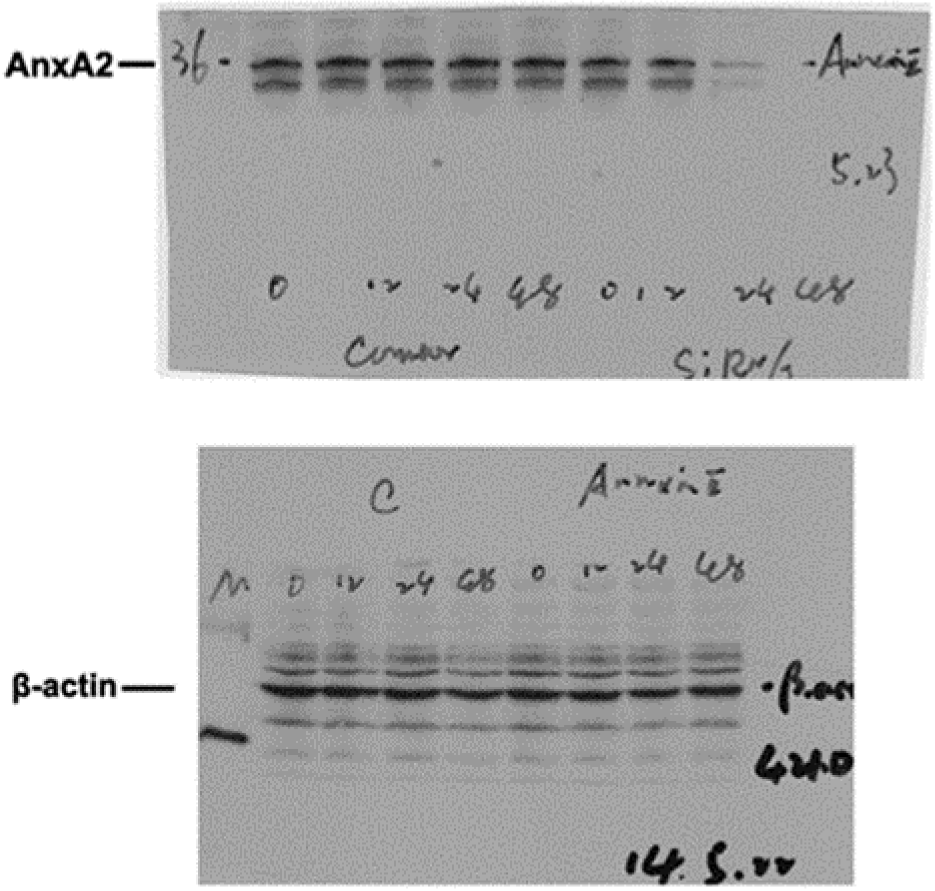

b

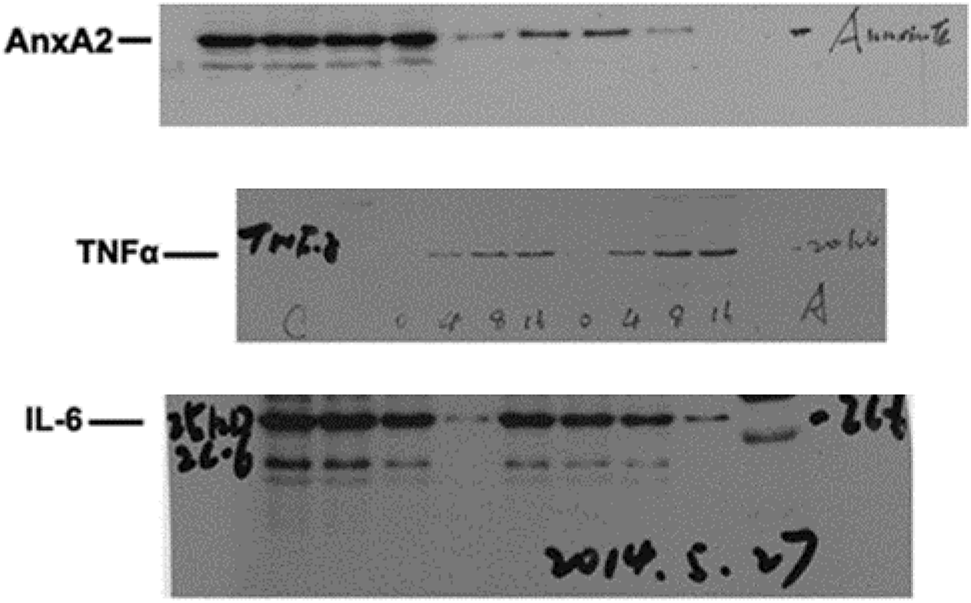

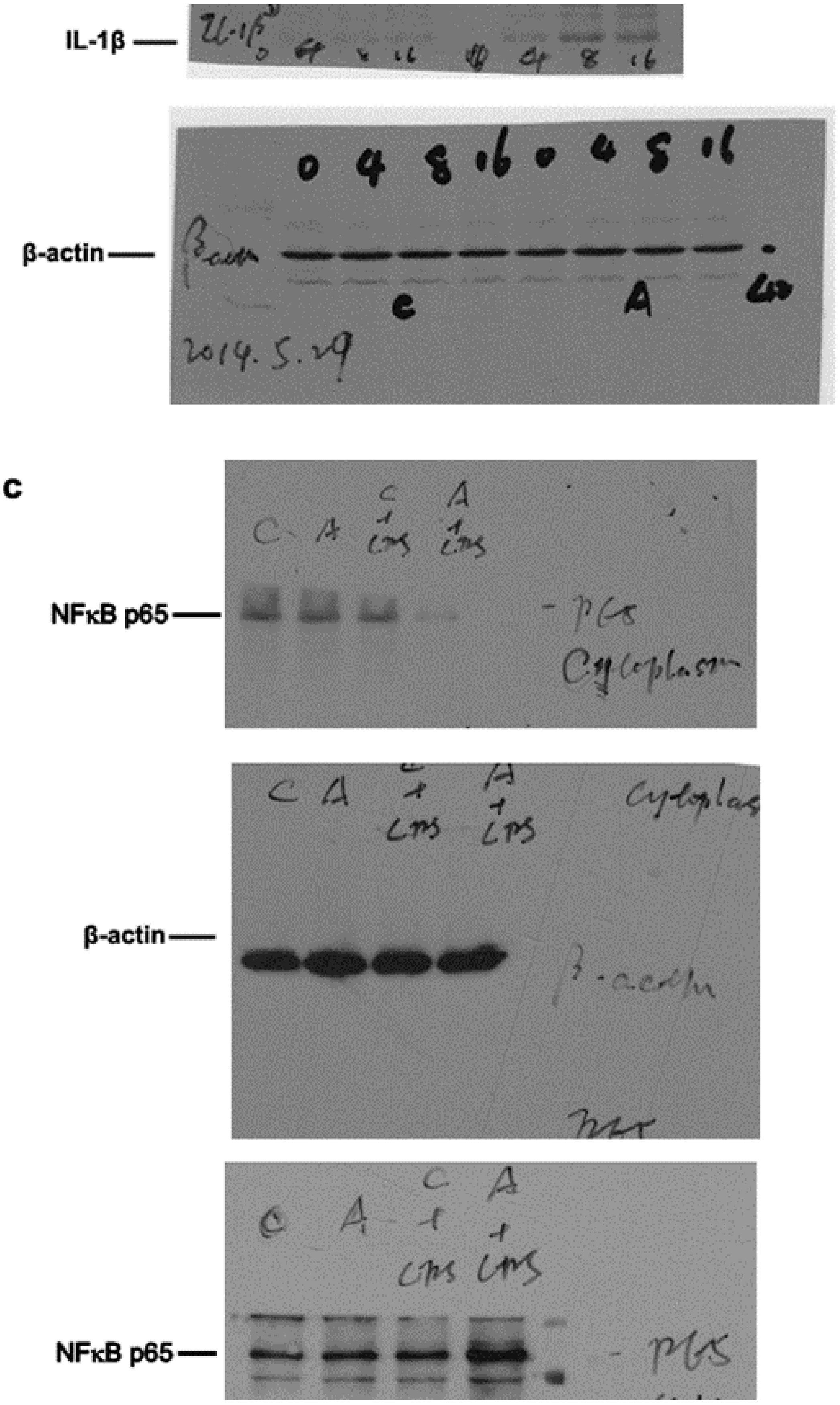
IL-1β
β-actin
C
NFκB p65
β-actin
NFκB p65

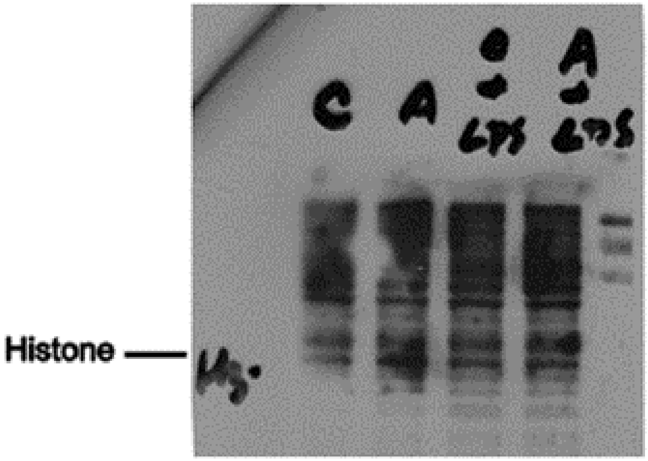

d

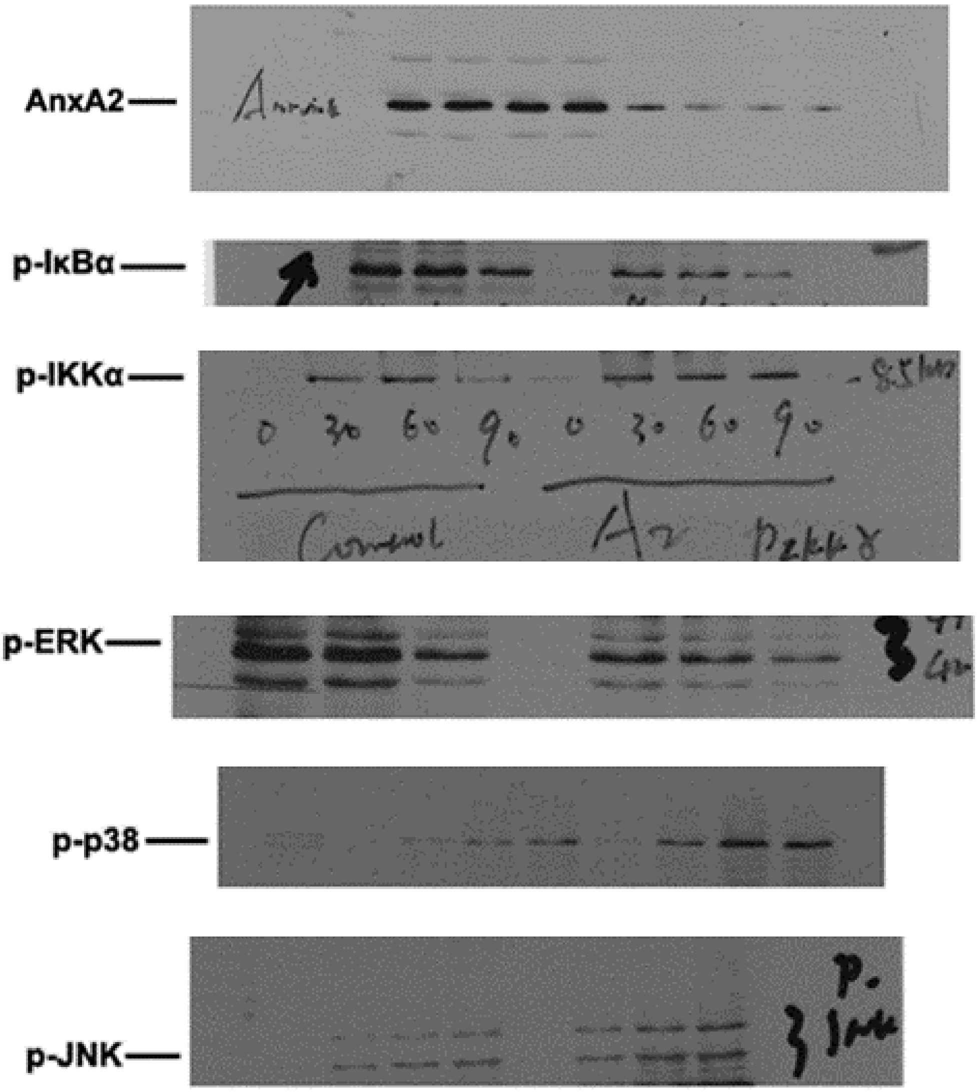

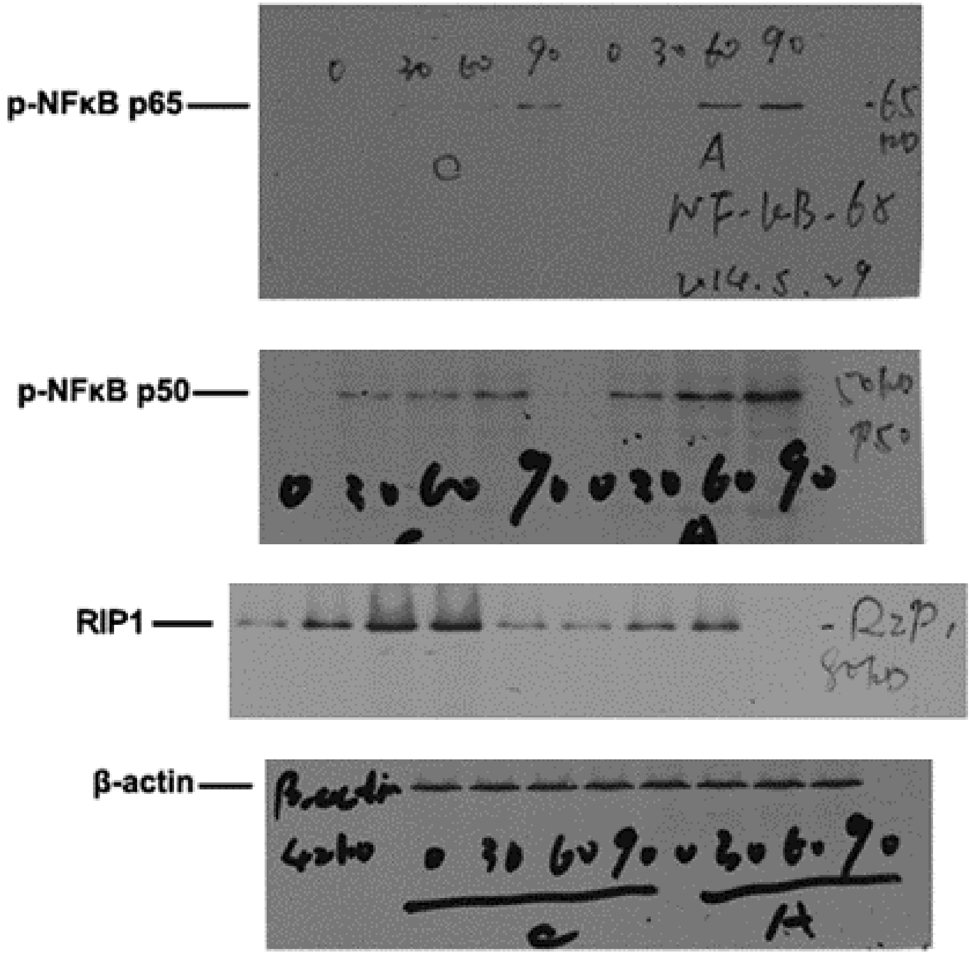

e

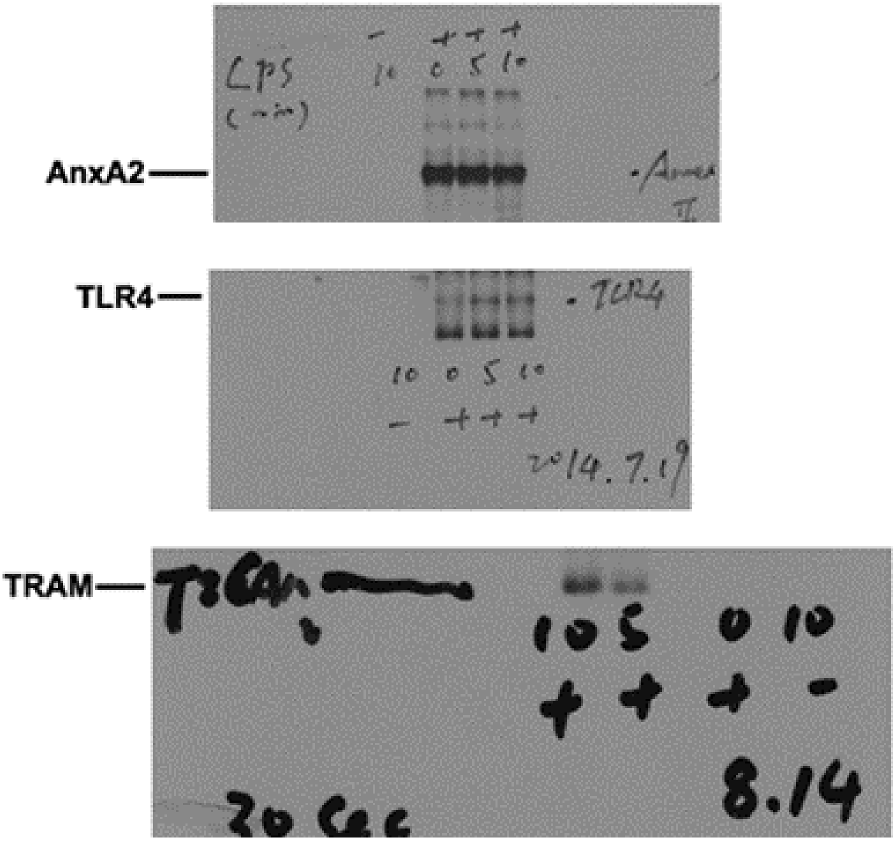

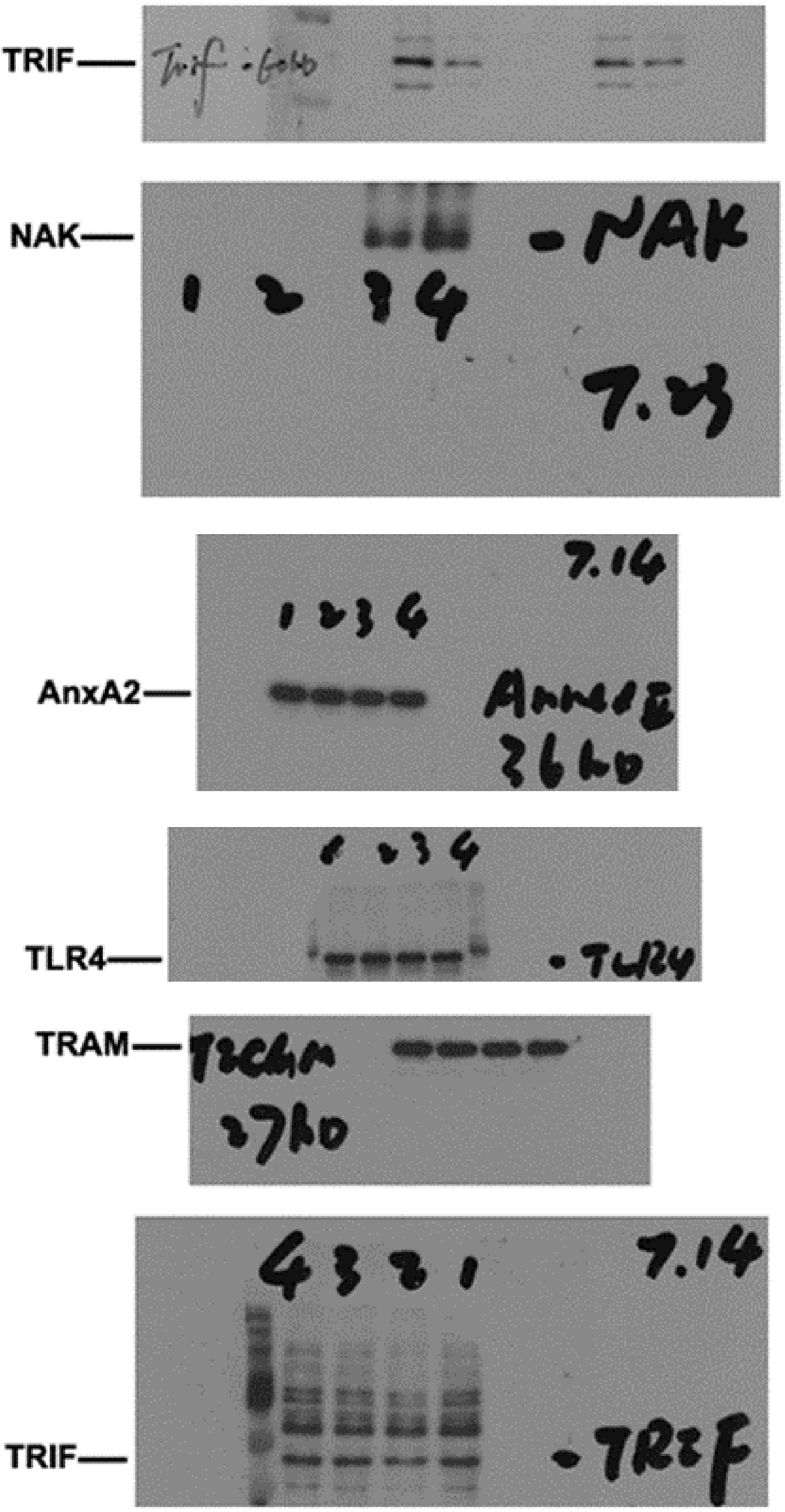
TRIF
NAK
AnxA2
TLR4
TRAM
TRIF

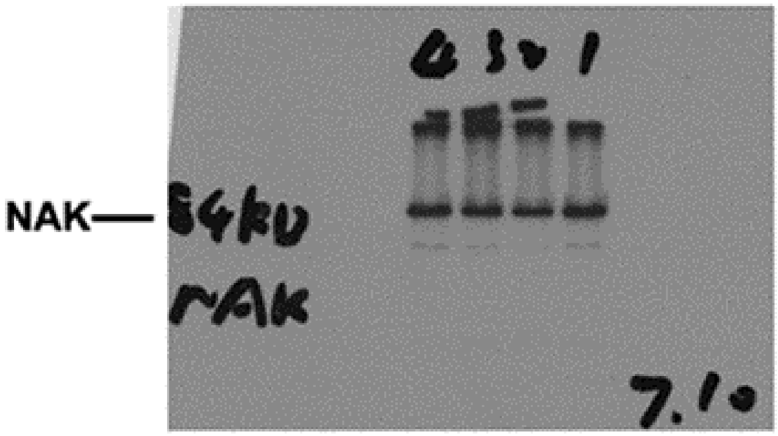

f

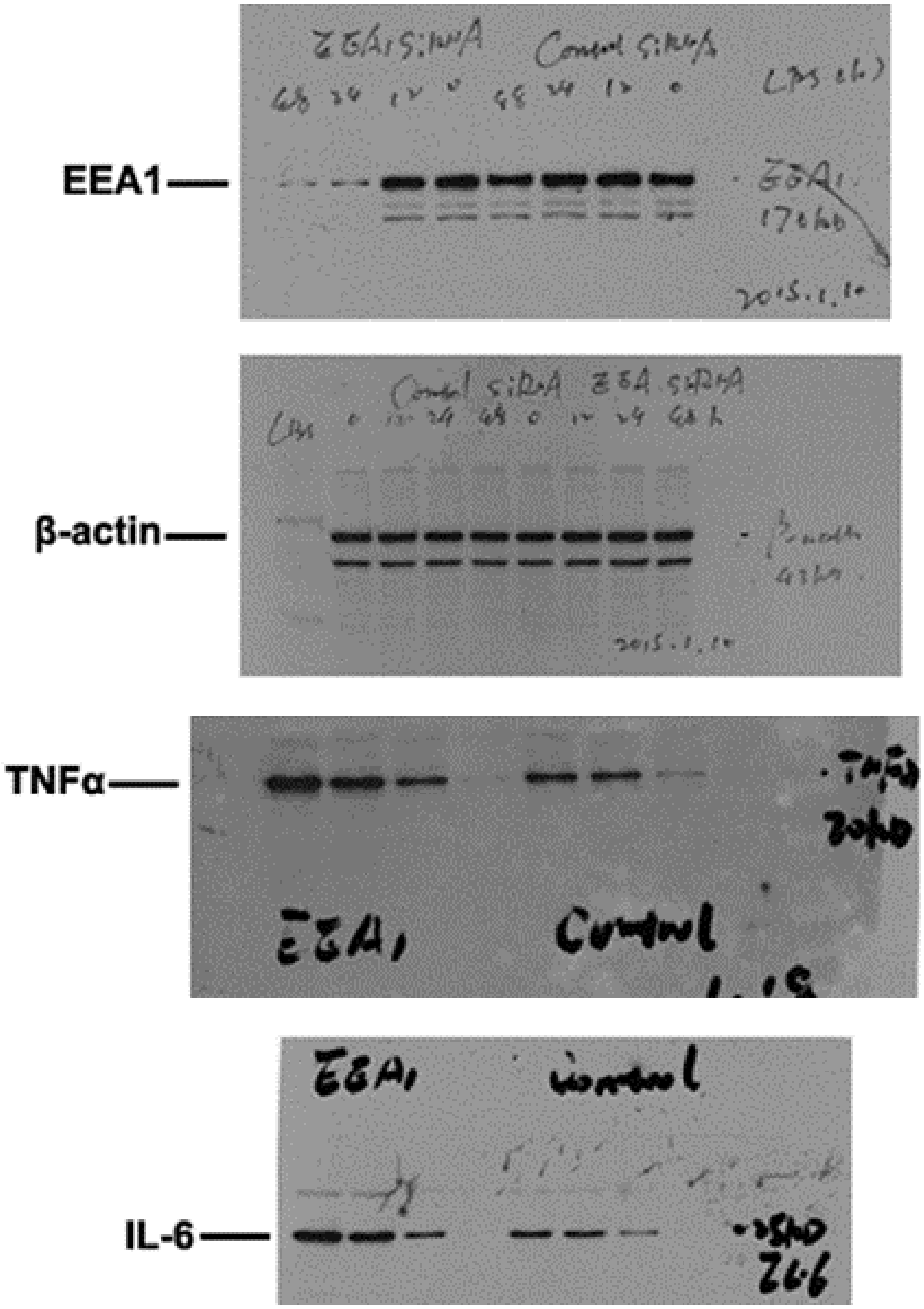

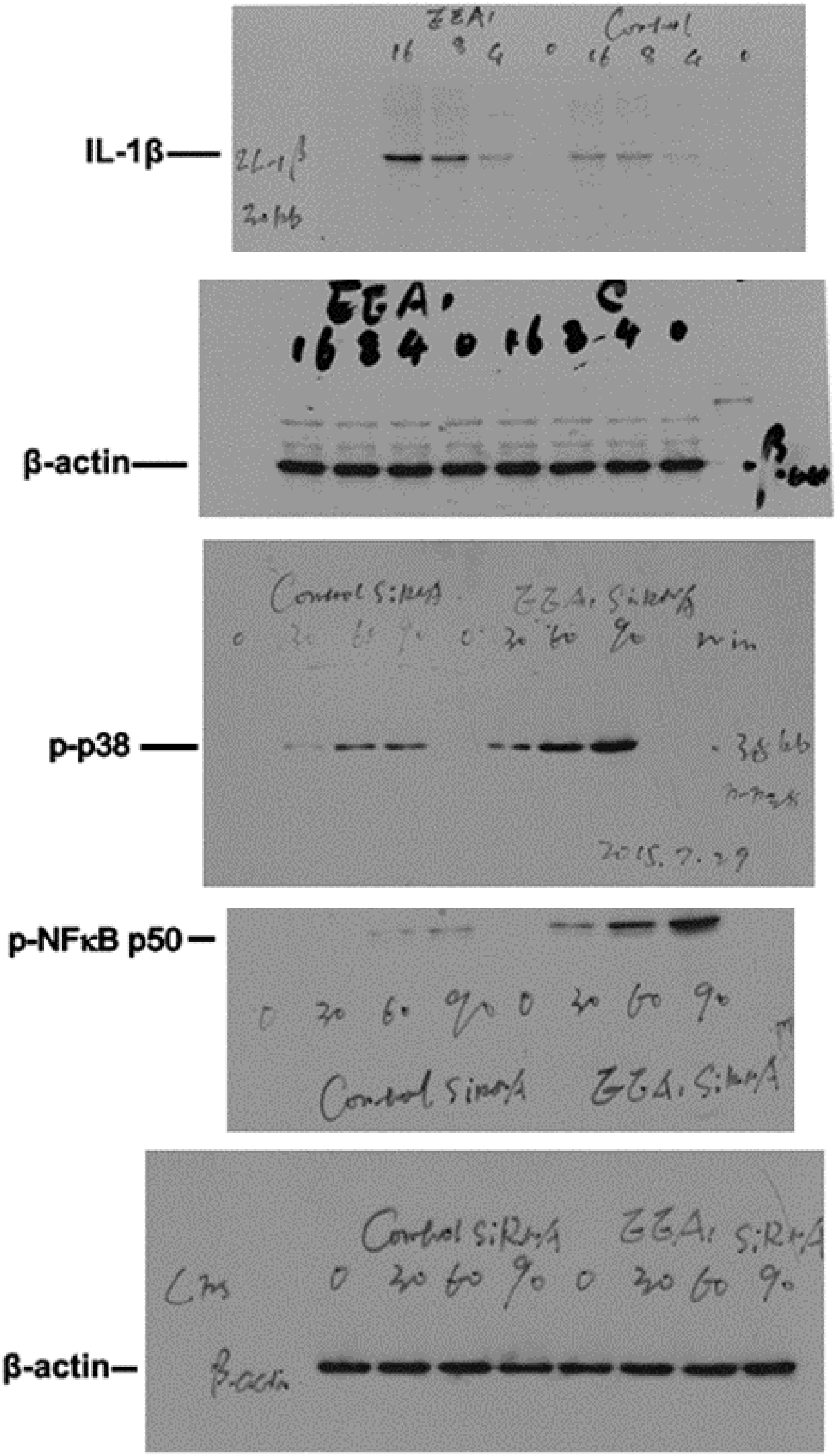
IL-1β
β-actin
p-p38
p-NFκB p50
β-actin

g

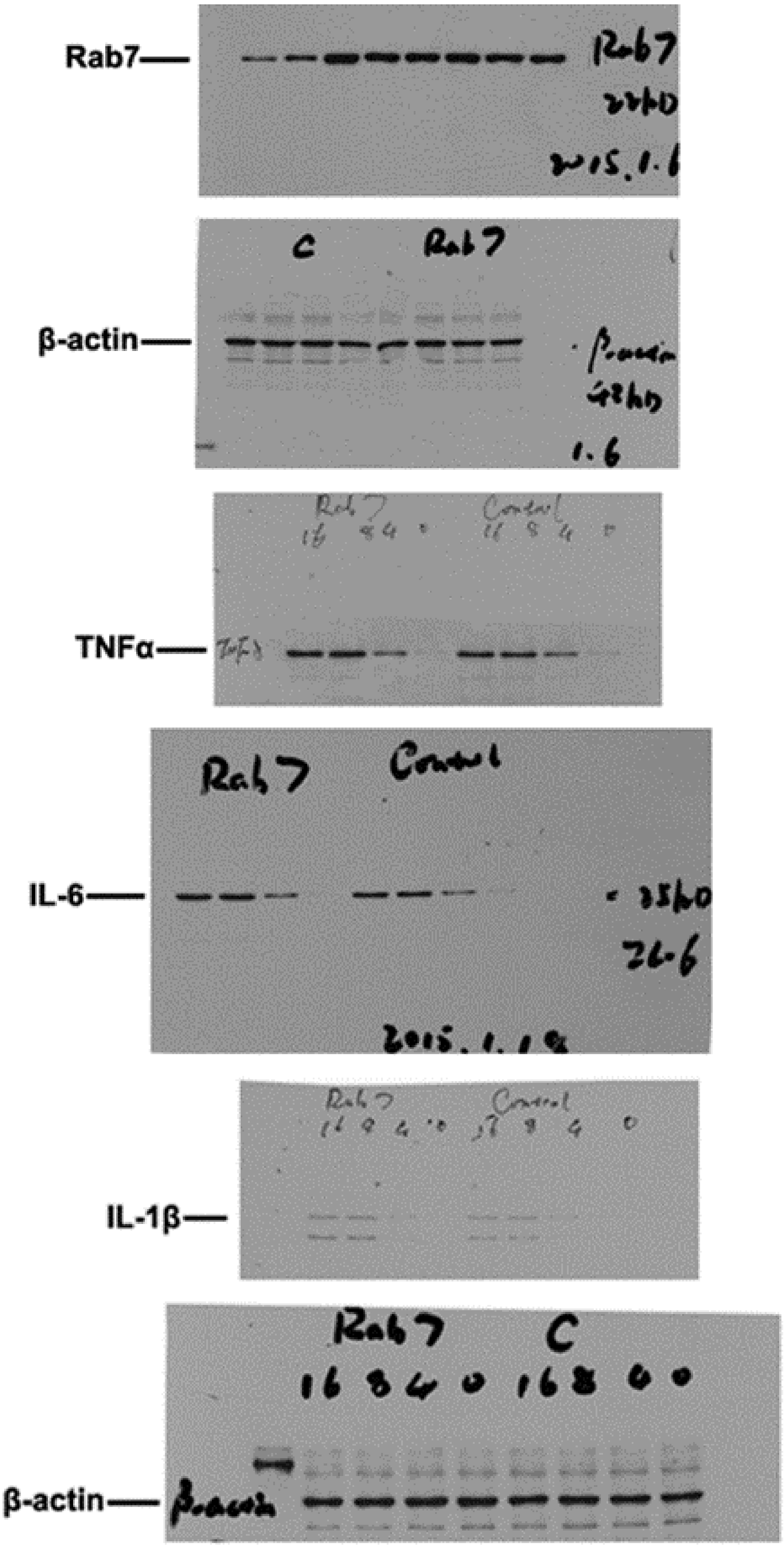

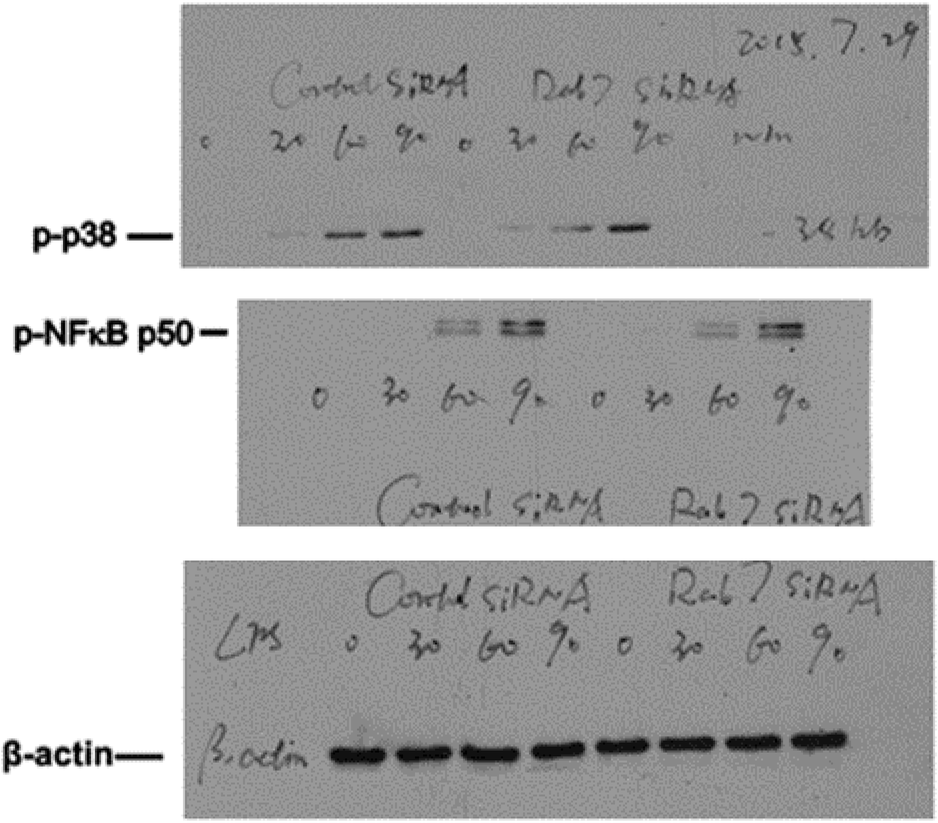

h

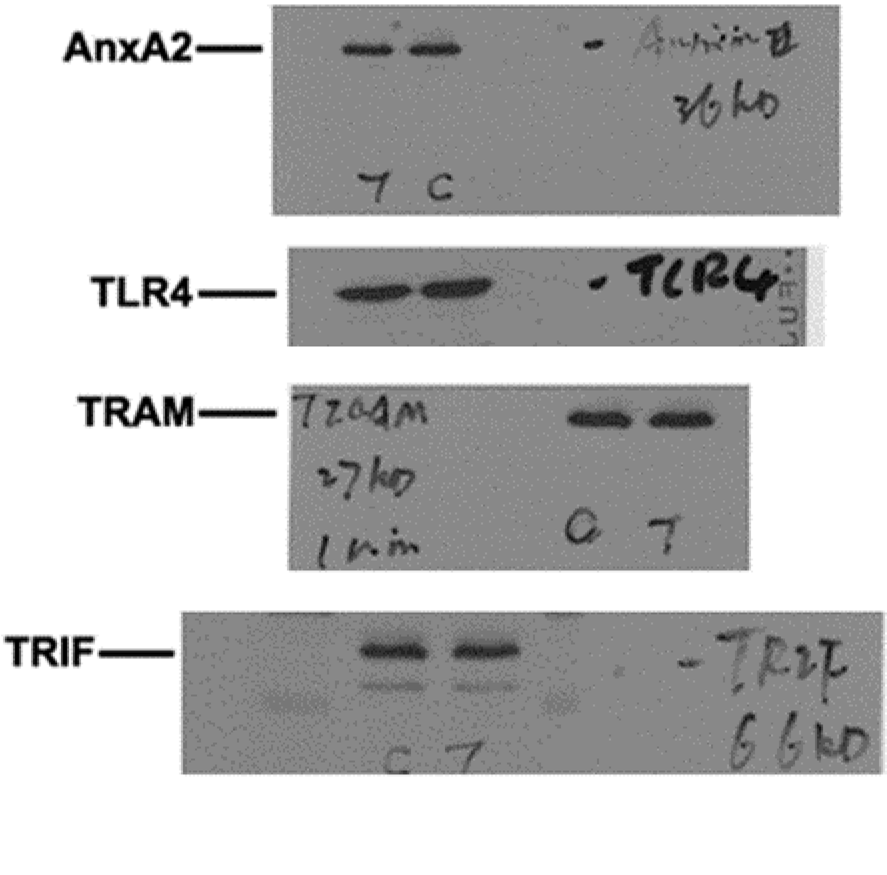

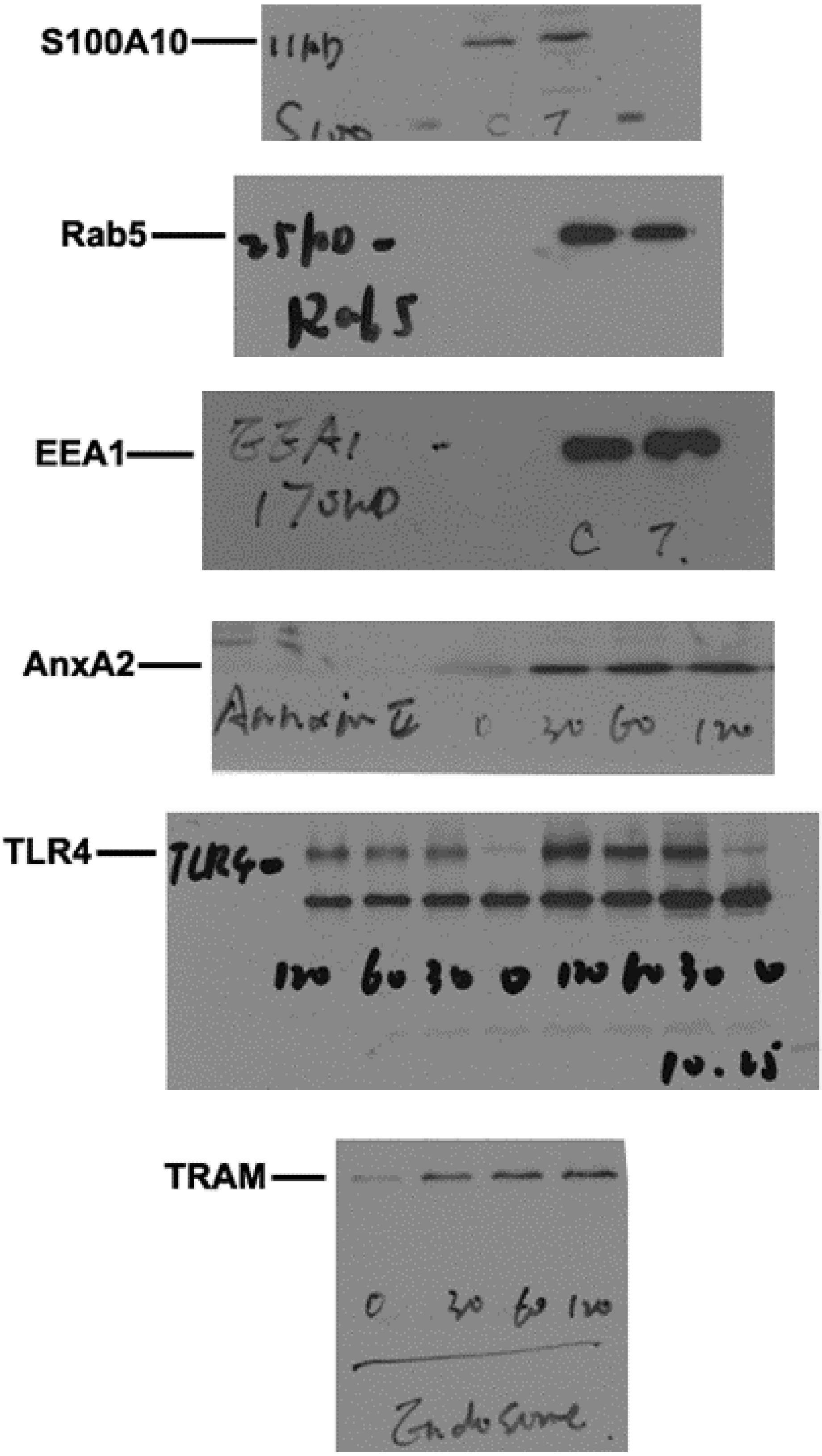
S100A10
Rab5
EEA1
AnxA2
TLR4
TRAM

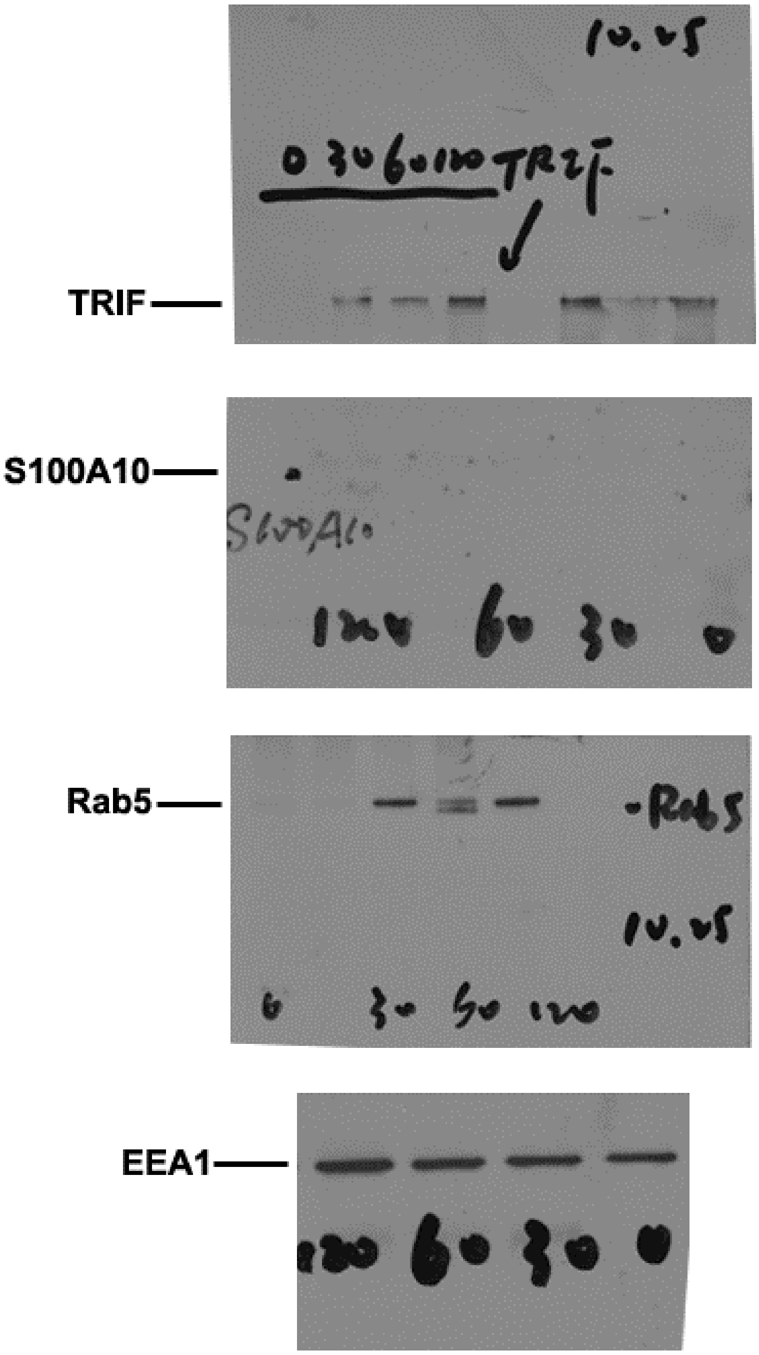
10.25
0 30 60 120 TRIF
TRIF
S100A10
S100A10
120 60 30 0
Rab5
Rab5
10.25
0 30 60 120
EEA1
120 60 30 0

i

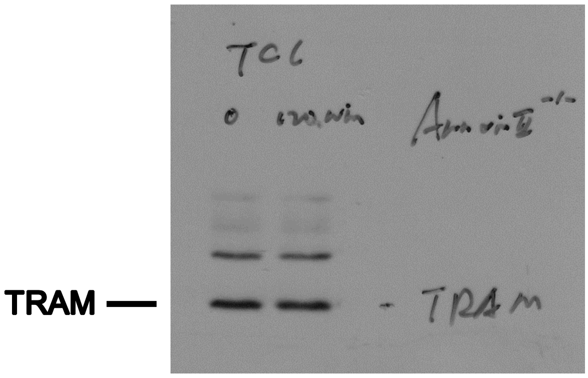
TCL
0 120min
TRAM
TRAM

TRIF —

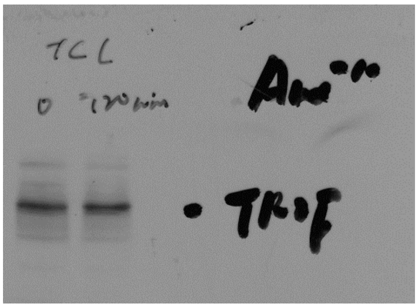

EEA1 —

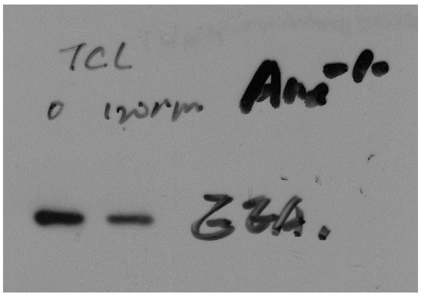

TRAM —

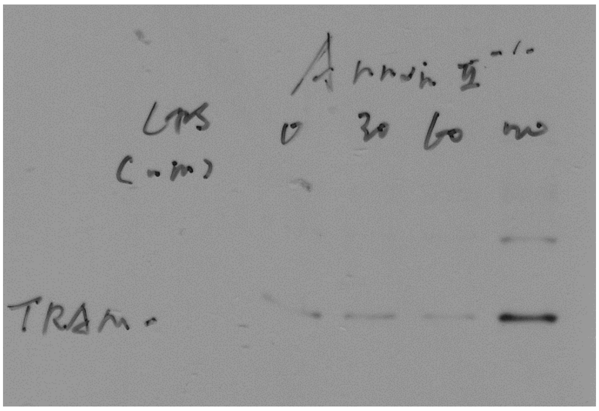

TRIF —

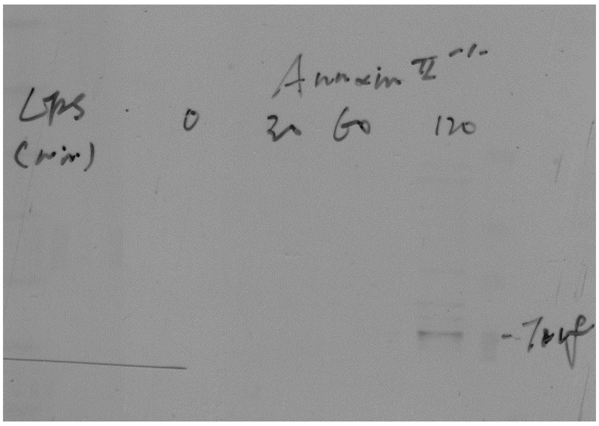

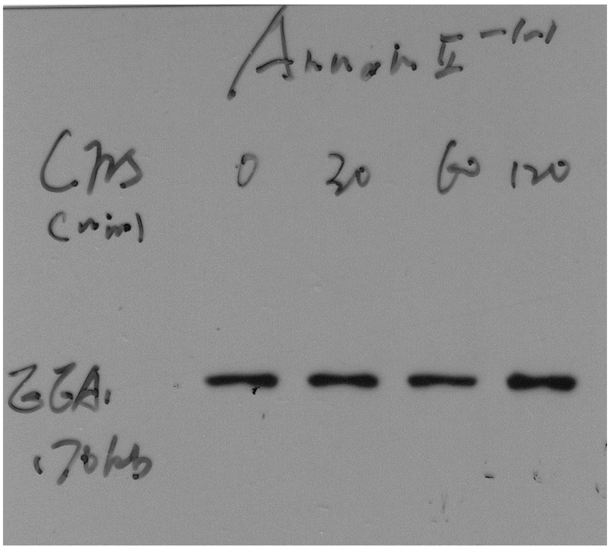

EEA1 —

j

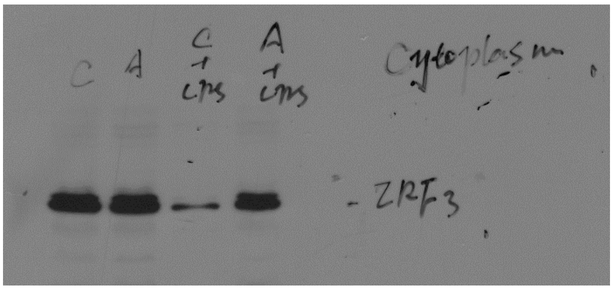

IRF3 —

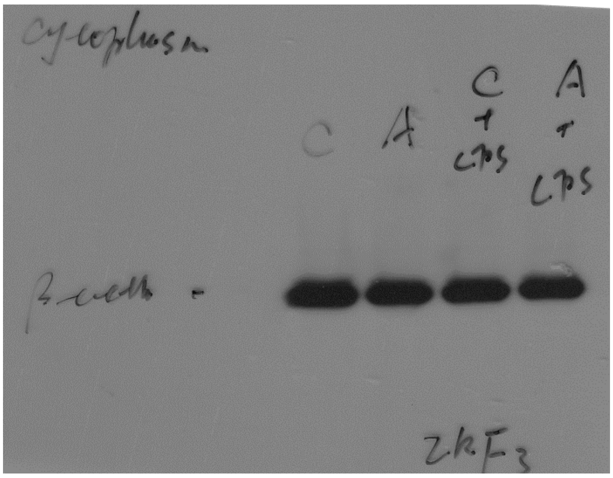

β-actin —

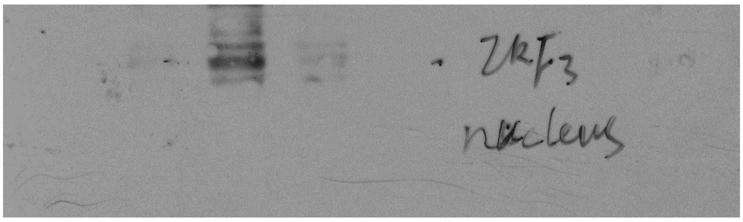

IRF3 —

Histone —

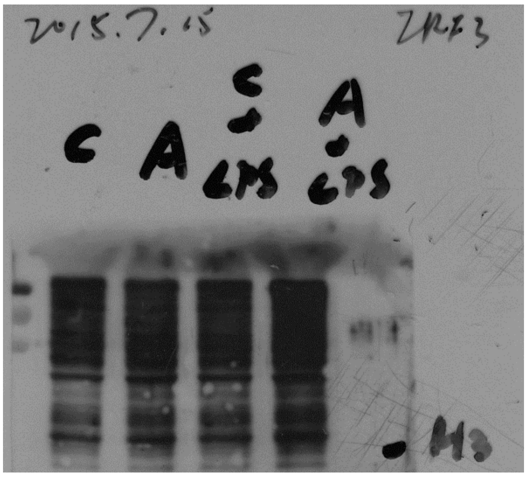

Supplementary figure S9. The full-length blots. (a) The full-length blots in Figure 3a. (b) The full-length blots in Figure 3b. (c) The full-length blots in Figure 3c. (d) The full-length blots in Figure 3d. (e) The full-length blots in Figure 4a. (f) The full-length blots in Figure 6c, d, e. (g) The full-length blots in Figure 6f, g, h. (h) The full-length blots in Figure 6i. (i) The full-length blots in Figure 6j. (j) The full-length blots in Figure 8c.
